# “What else to say?”–Primary health care in times of COVID-19 from the perspective of German general practitioners: An exploratory analysis of the open text field in the PRICOV-19 study

**DOI:** 10.1371/journal.pone.0282504

**Published:** 2023-03-17

**Authors:** Stefanie Stark, Marie Kluge, Emmily Schaubroeck, Felix Werner, Esther van Poel, Sara Willems, Marco Roos, Thomas Kühlein, Larissa Burggraf

**Affiliations:** 1 Institute of General Practice, Friedrich-Alexander University Erlangen-Nürnberg (FAU), Erlangen, Germany; 2 Department of Public Health and Primary Care, Ghent University, Ghent, Belgium; 3 General Practice, Faculty of Medicine, University of Augsburg, Augsburg, Germany; Uniformed Services University of the Health Sciences, UNITED STATES

## Abstract

**Background:**

The international collaboration study *PRICOV-19 –Primary Health Care in times of COVID-19* aims to assess the impact of the COVID-19 pandemic on the organisation of primary health care. The German part focuses on the subjective perceptions of general practitioners on primary health care and the impact of political measures during the second wave of the COVID-19 pandemic. Within this survey, the “open text field” of the questionnaire was utilised remarkably frequently and extensively by the respondents. It became clear that the content that was named needed to be analysed in an exploratory manner. Accordingly, this paper addresses the following question: What preoccupies general practitioners in Germany during COVID-19 that we have not yet asked them enough?

**Methods:**

The data collection took place throughout Germany from 01.02.2021 to 28.02.2021with a quantitative online questionnaire consisting of 53 items arranged across six topics as well as an “open text field” for further comments. The questionnaire’s open text field was analysed following the premises of the qualitative content analysis.

**Results:**

The topics discussed by the respondents were: insufficient support from health policies, not being prioritised and involved in the vaccination strategy, feeling insufficient prepared, that infrastructural changes and financial concerns threatened the practice, and perceiving the own role as important, as well as that health policies affected the wellbeing of the respondents. One of the main points was the way general practitioners were not sufficiently acknowledged for their contribution to ensuring high-quality care during the pandemic.

**Discussion:**

German general practitioners perceived their work and role as highly relevant during the COVID-19 pandemic. In controversy with their perception, they described political conditions in which they were the ones who contributed significantly to the fight against the pandemic but were not given enough recognition.

## Introduction

On top of being a biomedical problem, the COVID-19 pandemic presented itself as an overall social crisis of modern societies and exposed any previous structural problems in the respective healthcare systems [1–3]. The pandemic has destabilised and reconstructed longstanding systems and institutions, as well as posed a challenge to medical professional interactions all over the world. This pandemic thus presents itself not just as a global problem situation, but also as a crisis which exposes any previous structural problems in the respective systems, not least in the health care systems of the countries [4]. Such a prolonged crisis triggers state and political reactions, which are intended to protect the health care system. Regarding the COVID-19 crisis in Germany, it did not just lead to structural changes, but subsequently also to political measures designed to maintain the German healthcare system. This pandemic therefore posed organisational, structural, and content-related challenges to primary health care in the German general practitioners (GPs) practices [4].

Previous studies on epidemics and pandemics in primary health care prior to the COVID-19 pandemic can be found mainly in the context of SARS-CoV (pandemic 2002/2003), H5N1 (avian flu, epidemic 2006), H1N1 (swine flu, pandemic 2009/2010), and EHEC (Escherichia coli bacterium, epidemic 2011) [5]. Such studies serve as a starting point for further elaborations dealing in particular with outpatient care during and after this current pandemic. The research interests in those previous studies dealing with primary health care during a pandemic laid primarily in uncovering difficulties and challenges in primary care practices and what one can learn from these challenges [5].

The international collaboration study *PRICOV-19 –Primary Health Care in times of COVID-19*initiated by the University of Ghent (Belgium) aims to assess the impact of the COVID-19 pandemic on the organisation of care and the different dimensions of quality of care in GP practices in an international comparison [6]. In Germany, this study is being conducted by the Institute of General Practice of the Friedrich-Alexander University Erlangen-Nürnberg (FAU) and focuses on the subjective perceptions of the German GPs on primary health care and the impact of the pandemic political measures during the second wave of the COVID-19 crisis in Germany in February 2021. In the analysing process of this quantitative survey, it was noticed that the “open text field” was utilised remarkably frequently and extensively by the participating GPs. After a first look, it became clear that the further content that was named in this text field needed to be analysed in an exploratory manner. We took it as a necessity to conduct a qualitative analysis of the further comments in addition to the quantitative survey. Accordingly, the following research question arose: *What preoccupies German GPs during COVID-19 that we have not yet asked them enough within our questionnaire*?

## Methods and materials

### Study design and setting

Within this multi-country cross-sectional study, data were collected in 38 European countries. The Institute of General Practice of the Friedrich-Alexander University Erlangen-Nürnberg (FAU) is responsible for obtaining data for Germany. The ethical approval of this study was granted by the Ethics Committee of the Faculty of Medicine of the Friedrich-Alexander University Erlangen-Nürnberg (379_20 B), which did not raise any objections to the conduct of the study. Informed consent was obtained written through the online questionnaire. The data being transferred to the Institute of General Practice in Erlangen will not allow detecting the GP practices identity and is stored safely. All data is anonymised, and all raw data that could lead to the identification of the participants is permanently removed. Minors were not included in the study [6]. In this survey, the use of the “open text field” at the end of the questionnaire by the respondents was particularly noticeable, from which a large number of valuable answers regarding the content of the research interest of the PRICOV-19 study could be drawn [6]. Accordingly, this article will provide a qualitative analysis of the answers given from the German GPs to the open text field, which allows for a thorough determination of the concerns, perceptions, and attitude of the respondents.

### Development and validation of the questionnaire

The questionnaire was developed by Ghent University based on literature review and theoretical framework on quality of care [7, 8], patient safety culture [9] and discussions with GPs. Statistical validity was tested and guaranteed by Ghent University [6]. The questionnaire is included as an additional file in the study protocol [6]. The validated English translation of the questionnaire was provided to each research partner, to be translated into the country’s main language. When translating the questionnaire into German, a forward-backward method to guarantee contextual conformity was used. In addition, two German GPs had independently reviewed the translated questionnaire and adapted it where necessary.

### Questionnaire

The questionnaire consists of 53 items comprising seven topics: general information about the respondent and the practice, patient flow for COVID and non-COVID care, infection prevention, and information processing (e.g. dealing with new knowledge and protocols), communication with patients, cooperation and collegiality, as well as wellbeing of the respondent and self-care. To get a sense of the allocation, weighting, and content of the items in this questionnaire see Table 1.

**Table 1:** The questionnaire covers the following main content areas^a^.

Content area	Number of items	Sample items^b^
1. General information about the respondent and primary care	10	• *What is your position in this GP practice*? • *How many years of work experience do you have in primary care*?
2. Patient flow during and before the COVID-19 pandemic: a) Appointments b) Triage c) c. Safety management for routine primary care	a. 3 b. 4 c. 6	*a) Patients who made an appointment and where it is unclear whether they pose a risk of infection are called to verify this*. *b) In case the telephonic triage is performed by someone other than a GP in this GP practice and he/she needs support when assessing a call*, *he/she can rely on support from a GP*. *c) A patient with a fever caused by an infection other than COVID-19 was seen late due to the COVID-19 protocol*.
3. Infection prevention	5	• *In this GP practice*, *home care nurses are currently actively contacted when their patients are diagnosed with a major infectious disease (e*.*g*. *HIV*, *COVID-19*, *hepatitis carrier status)*. • *When patients are diagnosed with a major infectious disease the practice actively contacts the home care services to inform them about this*.
4. Information processing: dealing with new knowledge and protocols	2	• *In this GP practice*, *a fixed weekly time is provided in the agenda(s) of GPs for reviewing new guidelines or going through relevant and reliable scientific literature*.
5. Communication with patients	5	• *Does this GP practice have a brochure with information on COVID-19 to give to patients*?
6. Cooperation and collegiality	4	• *The guidelines imposed by the government on GP practices as a consequence of COVID-19 pose a threat to the good organisation of this practice*.
7. Wellbeing and self-care	4	• *The guidelines imposed by the government on GP practices as a consequence of COVID-19 pose a threat to the personal well-being of the staff in this practice*.

^a^ All questions were answered with a 5-point Likert scale using an agreement scale or could be answered with yes/no.

^b^ The selected examples listed here are representative of the remaining items and are only intended to provide an insight. Due to copyright, the complete questionnaire is not published.

Furthermore, another three topics with 10 items were added for the German questionnaire, which mainly deal with the context of GPs in Germany during COVID-19: implementation feasibility of standard care during COVID-19, acceptance of political measures/interference of politics in the GP profession, as well as structural changes in GP practice due to policy measures. The amended German questions were tested for comprehensibility using the thinking out loud method with four GPs and two non-GPs. The additionally added questions for Germany can be seen in Table 2.

**Table 2:** Added questions for Germany^a^.

Content area	Number of items	Sample items
1. Implementation feasibility of standard care during COVID-19	2	• *The care of COVID (suspected) cases cannot sufficiently ensure the care of uncomplicated diseases (e*.*g*. *back pain*, *urinary tract infection)*.
2. Acceptance of political measures / interference of politics and society in the GP profession	4	• *The measures taken by the government with regard to GP care to contain the pandemic have overwhelmed everyday practice*. • *The role of GPs has gained attention in society since the beginning of the pandemic*.
3. Structural changes in GP practice due to policy measures	4	• *The local structures of medical cooperation (e*.*g*. *interprofessional exchange*, *substitute organisation) have changed positively as a result of the pandemic*.

^a^ All questions were answered with a 5-point Likert scale using an agreement scale.

### Open text field

At the end of the questionnaire the participants were offered the possibility to add any further comments or suggestions and feedback in an open text field. The open text field was the last item of the questionnaire and the punctuation limit was 500 words. The open text field was phrased as follows:

*“Thank you very much for your participation. You have gone through all the questions of this questionnaire*. *Lastly, we would like to hear if you have any additional comments or suggestions for us. All feedback is welcome and can be entered in the text box if you wish.”*

The open text field was included in the questionnaire of all countries participating in the study. It gives the respondents the possibility to mention what was not already asked within the questionnaire and allows for a concrete and precise determination of, e.g. concerns or subjective perceptions of the participating GPs. Open-ended questions or open text fields in quantitative surveys are used to explore, explain, and/or reconfirm existing ideas [10]. As the analyses in the different countries are still ongoing we can assume that there were differences in filling out and using the open-text field: from frequent use to hardly any depending on each country. In Germany, we considered 85 completed open-text fields with an average of 300 words as valuable in terms of content to be very interesting and relevant to provide a qualitative approach to analyse those answers given.

### Recruitment and sample

German GPs were invited to fill in an online questionnaire. The recruitment in Germany took place throughout the second COVID-19 wave in February 2021 (from 01.02. to 28.02.2021). The requirement for the international study comparison was 200 GP practices (n). Ghent University based requirements on specific ratios per country: countries with more than 10,000 GPs should recruit at least 200 practices. We assumed a predicted response rate of 10% for Germany. The questionnaire was directed to GPs, specialists for internal medicine, and GP trainees who work in single or joint practices in rural or urban settings. In the following, all participants will be referred to as GPs for better readability. The questionnaire was sent out via e-mail and could be completed online. The participants were able to access the questionnaire through the link in the email which guided them to the online platform REDCap [11]. The informed consent form and data protection notice preceded the online questionnaire and had to be agreed to. Filling out the questionnaire took about 20 to 30 minutes. We recruited nationwide in a snowball procedure (in order to gain quick and broad access to the research field) via anonymised e-mail dispatch lists from the primary care institutes’ teaching and research practices (FAU Erlangen), other primary care institutes (throughout Germany), German primary care practices networks, as well as the Bavarian General Practitioners’ Association. No incentive was given to the participants and no reminders were sent to enhance response rate. All data were collected anonymously and stored on the secure Ghent University data servers [6]. By using the method of the snowball system, we were able to send out the survey link to 1,710 GPs throughout Germany. In order to be able to track the data collection and the targeted N, weekly updates were sent from the REDCap server regarding the already completed questionnaires. Accordingly, a final n of 349 practices was formed who filled out the questionnaire (estimated response rate: 21%).

## Analysis

The analysis loosely follows the basic assumptions that the COVID-19 pandemic as a crisis [4] made clear that previous structures can no longer be held onto and need to be restructured in primary care. Thus, it will be explored how the German GPs perceived the health policy measures and the changes on primary care in general and how this influenced their perception of practitioners of a medical profession in times of such a crisis.

Any comments or suggestions received were analysed following the principles of the qualitative content analysis which is based on a category system that is hierarchically structured into main and subcategories [12]. Additionally, the intersection of certain categories is analysed as it gives further insights into the importance of specific topics. In advance, it was necessary to specify which parts of the communication model the analysis intends to make a statement on (i.e. the addressee or the producing entity). In this context, the analysis intends to make a statement about the producing entity, i.e. the participating GPs, and their subjective perception of the matter at hand. In this analysis, five main categories (see Table 3) were established inductively in an initial sighting by two researchers of the Institute of General Practice (FAU) independently of each other. Using this method of the independent four-eyes-procedure, the data were sighted and coded one after the other by these scientists and then discussed, evaluated, and validated in a consensus discussion with two further scientists of the Institute. The same procedure was followed with the results as they were checked for plausibility. The latter procedure illustrated the need for a subdivision of the categories, as the answers covered a myriad of topics. The subcategories were then established in a second, more thorough sighting which also included classifying the answers in the respective categories. The answers of the open text field were categorised as shown in Table 3.

**Table 3:** Category system and coding guidelines.

Category	Definition	Subcategories	Guidelines for Coding
I. Policies and Health Care System	• negative and positive aspects of the decisions made and measures implemented by the government(s) • impact of policies on the health system • support and communication from the government(s)	• no prioritising • support • health policies and information	*keywords should be mentioned concerning the defining principles* *every aspect that can be or has been influenced by policies will be regarded*
II. Cooperation	• view of the cooperation not only between GP colleagues but also in terms of the exchange between different specialists, professions, academia or scientific research, government(s)	• preparedness • communication	*keywords should be mentioned about the defining principles* *critique*, *as well as praise for colleagues/members of the respective party*, *will be regarded*
III. Concerning the Practice	• structural changes that had an impact on not only the GP but the practice team as a whole • organization and implementation of new measures • financial aspects	• infrastructural changes • vaccination strategy • financial concerns	*keywords should be mentioned about the defining principles* *remarks concerning the GP as an individual (i*.*e*., *removed from the practice context) will not be regarded*
IV. Role and Self-perception	• the subjective definition of the professional role in general • as well as the own personal role as a medical professional in times of a crisis • perception of one’s own conduct	• role • perception	*keywords should be mentioned concerning the defining principles* *remarks concerning the self-perception and that of the role regarded*
V. Concerning the Individual	• everything that goes beyond the person as a GP and focuses on the participant as a human entity • emotions and hardships • psychological implications	• mental wellbeing • individual problems	*keywords should be mentioned about the defining principles*

Here, only those comments were considered for further evaluation that provided answers of value in terms of content within the framework of our explorative approach. Answers or comments on the questionnaire or the study itself etc. were disregarded. Therefore, the remaining category *Others* was not of special interest apart from the solicited feedback on the questionnaire in general and therefore no further part of the analysis. For transparency reasons, it is nonetheless attached to the S1 and S2 Files. Regarding the quality of the answers or comments given in the open text field, it can be stated that they range from 10 characters (the shortest) to 150 characters (the longest). The answers are not in the form of bullet points, but are formulated as entire sentences that often pick up on a particular keyword in the questionnaire or link several together. It is striking—and this is also the special characteristic of this study—that in the open text field, new points were addressed that were only partly dealt with or not dealt within the previous questionnaire of this survey at all. As the coding rules called for specific keywords, an index (see S1 and S2 Files) was formed that marked the respective words and gives a quantitative summary of the keywords mentioned. The keywords were kept rather general, such as *changes*, *profession*, *influence*, *decisions*, *challenges*, *role*, *policy*, *support*. In a subsequent step to categorising all the answers given, the categories were analysed individually and in relation to their respective subcategories that were built to warrant concrete statements about each topic. The answers from the open text field were extracted from the data set (see S3 File) via IBM SPSS Statistics 28, recorded and collected in a separate Microsoft Office Word document, prepared in tabular form, and categorically processed via paper-pencil-principle and the index function in Microsoft Office Word (see S1 and S2 Files). As the data was in German, all data was translated to English (S1 File). Each statement was marked numerically as text segment (e.g. T1, T2, etc.), which serves as a citation reference in the aforementioned S1 File.

## Results

The following results are presented according to the category system and coding guidelines which can be seen in Table 3. Many of these comments contain criticism or appear emotional, which is reflected, for example, in the capitalisation of specific words. However, the participants do not only refer to certain questions in the questionnaire, but to the overall topic that the questionnaire deals with, namely the impact of the COVID-19 pandemic on primary health care in Germany.

### Sample characteristics

In total, 85 participants used the open text field. The characteristics can be seen in Table 4.

**Table 4:** Cohort characteristics.

		OVERALL		OPEN TEXT FIELD	
		n	%	n	%
**Participants**		349	100	85	100
**Position/Function**	GP	208	59.6	70	82.4
	Internists as GP	43	12.3	13	15.3
	GP trainees	8	2.3	1	1.2
	Missing	90	25.8	1	1.2
**Work experience in years**	< 5	30	8.6	6	7.1
	5 - < 10	26	7.4	3	3.5
	10 - < 15	33	9.5	12	14.1
	15 - < 20	44	12.6	13	15.3
	20 - < 25	48	13.8	20	23.5
	25 - < 30	34	9.7	16	18.8
	30 - < 35	23	6.6	10	11.8
	35 and more	12	3.4	3	3.5
	Missing	99	28.4	2	2.4
**Practice type**	Single	218	62.5	71	83.5
	Group	111	31.8	14	16.5
	Missing	20	5.7	-	-
**Practice location**	Rural	91	26.1	41	48.2
	Small Town	95	27.2	11	12.9
	Urban	76	21.8	32	37.7
	Missing	87	24.9	1	1.2
**Practice payment system**	Private	38	10.9	8	9.4
	Health insurance/private	221	63.3	76	89.4
	Missing	90	25.8	1	1.2

### Open text field

#### Policies and health care system

The GPs most frequently referred to the *pandemic policies* within the German *health care system* in the open text field.

*Insufficient support from health policies*. The majority of the respondents referenced to the *insufficient support* from health policies. Those answers were overshadowed by the sheer number of emotional viewpoints that established a general negative sentiment toward the way political stakeholders had managed the crisis: GPs explained that this takes a toll on the professional life as well as the mental health and described themselves as left feeling "abandoned", "sad and frustrated", and not knowing "how to go on" (T4, T8). Concerning the German *health policies* and the *information* flow from the government and the health system during this pandemic, the GPs found that the "political coordination is an absolute disaster" (T11) and had an even more negative impact on the daily practice life and the exchange with patients than the virus itself (T10). In a similar vein, the "flood of weekly, even daily information and the nonsense spread in the press regarding Covid weighs more on my mind than the treatment and handling of patients" (T18). Implementing the weekly or daily changing requirements "ties up a lot of energy and time, which is then lacking for direct patient contact" (T21). The fact that these measures were superimposed and decided on by often external authorities that are usually not concerned with the medical field furthermore exacerbated the common resentment (T13). This discrepancy in the policymaking and the experiences of the GPs is criticised explicitly: "As GPs, we experience a discrepancy between statements, figures, political action and experience on the front line. The psychological/physical, social damage, especially to the young, is frightening, the measures disproportionate” (T26). A reason for this discrepancy between authorities and practicing doctors or GPs could be the non-existent communication: "Pitiful is the ignorance of the people even after one year of pandemic. The state as an educator has failed […] Communication gets a straight five (Grade 5 equals the mark “insufficient” in Germany)" (T27). One political stakeholder that was highly criticised for its shortcomings during the pandemic was the “Association of State Insurance Physicians (KBV)”. The participating GPs said that their support was insufficient or their measures "extremely complicated" (T17, T14).

*GPs felt not prioritised in the vaccination strategy*. Regarding the vaccination strategy, many felt as though the GPs "were not extensively prioritised" (T2). They perceived "an early and speedy vaccination" (T5) as vital means to grant "great relief for all practice staff members" (T5). Not having been *prioritised* in the vaccination process was furthermore perceived by some to be a symptom of an underlying bigger problem, namely the fact that society and policymakers perceive GPs as "second-class doctors" (T3). The field of general practice altogether seems to have always been "neglected, unappreciated and completely underpaid" (T6). A problem that also seemed to linger, in turn, was the implementation of a fast and steady *vaccination strategy*. Besides the individual dissatisfaction with the missing prioritisation for GPs, the lack of vaccines was lamented. Interestingly, the GPs responsibility toward the patients was of main concern, as the poor organisation of the vaccination process "in general and especially for elderly patients cared for at home" (T49) was seen critically. Not just the insufficient quantities of vaccines were criticised, but also the late introduction of the vaccinations (T48) and, together with a felt responsibility, this presented a source of concern for the GPs: “I fear that the Covid vaccinations will be delegated […] to the primary care practices too soon by the politicians after the appropriate vaccines have been approved and that we will once again have to explain ourselves to our patients, through no fault of our own, due to a lack of sufficient vaccine quantities…" (T48).

#### Cooperation between different actors in the health system

The *cooperation* between several different actors was also repeatedly mentioned with link to *preparedness* and *communication* in this pandemic.

*GPs felt insufficiently prepared and excluded*. The *cooperation* under colleagues or regional practices was not among those categories frequently referred to, yet one aspect that was also highlighted is that the GPs value their colleagues for great work they’re doing: the "willingness of most […] to roll up their sleeves and lend a hand" (T31) made it easier to deal with the pandemic’s implications. GPs perceived themselves and their colleagues as a cohesive unit in the pandemic, having each other’s backs and sticking together. Some participants used the opportunity to deal out criticism that they were not sufficiently *prepared* for the pandemic in their eyes (T41). The *cooperation* between policymakers and primary care was perceived to be insufficient and the GPs saw themselves neither noticed nor involved enough (T38). Furthermore, the *communication* between medical professionals, scientific researchers, and political agents was seen critically. In the respondent’s view, the federal and state governments relied too heavily on "the advice of individual experts of their choosing" (T39) rather than listening to the medical experts in primary care–the GPs.

#### Concerning regarding practice itself

The GPs also mentioned a lot of aspects that influenced *the practice* itself, like *infrastructural changes* and *financial and economic concerns*.

*Infrastructural changes threatened the practice routine*. In general, *infrastructural changes* and adjustments threatened the seamless practice routine. Many admitted that their practice could only "be organised so well" (T43) because they already had a separate space or rooms that could be used for treating possible COVID-19 patients. Others had to adjust their infrastructure to separate infected and non-infected patients (T42). Another adjustment that was made were specifically implemented consultation hours that were aimed at possible COVID-19 cases. Some of these measures were implemented to "protect the ’normal’ patients and give them the confidence to go to the GP" (T42). Mirroring the sentiment that the GP should remain the first point of initial contact of the patients and is aware of their importance for the patients and the stability they provide for the latter. These adjustments took some time and were facilitated by the good cooperation of the patients and the teamwork within the practice (T47).

*Financial and economic concerns*. Another aspect that influenced the practice routine were *financial concerns*. The COVID-19 pandemic induced decline in patient numbers which caused “new financial ‘imbalance[s]’” (T51). To mitigate the "considerable financial and economic risks for the practices" (T52) caused by political bodies through lockdowns, there was a call for an adjustment in the remuneration of medical services. The so-called phone consultation was previously not considered as compensable (T53). Here, an intersection with the underlying self-perception of the GPs became visible: daily free phone calls for a duration of up to four hours is, apparently, “unworthy of our profession” (T53).

#### Role and self-perception of the GPs

Another category established addresses how the GPs perceived their *professional role* and themselves during the pandemic, and contains the subcategories of *role* and *perception*.

*GPs perceived their role as important*. It became clear that the participating GPs perceived their *role* as vital and important during this pandemic. Understanding themselves as the initial point of contact for most patients, the goal was mentioned to provide "support and peace of mind" to the patients and contribute the part expected from the profession to cope with the pandemic (T29). The GPs said that dealing with the pandemic is "part of the job" (T29). However, here the role as GP was assessed in relation to the societal expectation: "The role of GPs is not strong enough" was the statement of concern (T4). The same participant also expressed concerns about how the GPs were not sufficiently prioritised in the vaccination strategy and in terms of the policy measures implemented. Here, it becomes evident that the producing entity perceived the low societal status of the GP role as inextricably linked to the political decisions made on the profession’s behalf. In addition, participants generally stated that dealing with the pandemic, as dangerous and challenging as it may be, is "a fundamental medical task" (T32), which in turn demonstrates their *self-perception* of their profession. This can be explained by the fact that pursuing a medical profession is perceived more as a social form than as a mere occupation. The discourse surrounding the profession is vital in the perception of the former itself in society, as well as for the perception of the GPs carrying it out. The self-perception, in turn, also has an influence on the development of an occupation into a profession, which is held to academic standards and obtains certain responsibilities and a commitment to the common good of society. However, it is lamented that this axiomatic fulfilment of the profession’s task was not sufficiently appreciated through financial support or political appraisal (T34, T35).

#### Concerning regarding the GP as individual

The last category addresses the subcategories of *mental wellbeing* and *individual problems*.

*Policy measures affected the wellbeing*. Not many answers were given but the impact the "additional burdens" such as new implementations due to health policy measures, bureaucracy, and other changing requirements had according to our respondents an impact on the *wellbeing* (T55): the producing entity alluded to the impact the pandemic had on their colleague. In the latter’s specific case, burn-out had rendered them incapable of working, leaving the participant in charge of the practice. This aggravated the working situation and ultimately led to the consideration of entirely "GIVING UP the practice” (T55; emphasis in original).

## Discussion and conclusion

### Discussion

In summary, two concise themes can be identified from the results: Firstly, the GPs who participated in the German part of the Pricov-19 study used the open text field to address things that, in their opinion, went wrong during the second wave of the COVID-19 pandemic in Germany. In particular, they refer to the insufficient appreciation of their role and criticise the guidelines given by the politicians (categories: policies and health care system/role and self-perception). Secondly, the results show that GPs take their role in the pandemic seriously and demand that it should be emphasised by clearly reading out that the role of GPs was not strong enough from their perspectives. This is particularly apparent in the COVID-19 pandemic and goes hand in hand with the previous statement of dissatisfaction with political measures. Here, too, it was criticised that the GPs did not feel sufficiently prepared during the pandemic and that the implementation of the measures and requirements was only possible with sufficient infrastructure (e.g. additional treatment room to be able to offer infection consultation hours). The GPs would also have liked to be more involved in the vaccination process at an earlier stage in order to be able to contribute their previous experience (especially with regard to the implementation of vaccinations and the planning of the number of vaccinations etc.).

In other studies that quantified these issues, the majority of GPs said they were very poorly or poorly prepared for this pandemic and that the political measures to mitigate the pandemic were inadequate [13]. Several studies have been conducted in the medical field on the impact of the COVID-19 pandemic on the core functions of primary health care who come to the same point of view [14–16]. On the German national level, the *COVID-GAMS* study and the *COVID Practice Survey* examined how the pandemic shaped and changed the daily work life in general practices [17, 18]. Here, too, the majority of respondents rated the relevance of the ambulatory sector for coping with the COVID-19 pandemic as very relevant. Furthermore, the results in these studies showed that German GPs and their practice teams were under high pressure during this pandemic and that the structures, the organisation, as well as the processes in the GP practice have changed since the beginning of the COVID-19 pandemic. GP emergency services were used for COVID-19 testing, vaccinations should or will henceforth be given in GP practices, and infectious disease consultations are implemented. These studies pursued the goal of capturing and presenting the current state of the pandemic as well as primary care with all its facets in order to design solution strategies for the current, but also for possibly following pandemics [17, 18]. In addition, it must be added that the "open text field" was also often used in those studies just mentioned. This shows that the COVID-19 issue does not pass by GPs without leaving a trace and that there is a corresponding need for discussion and exchange as well as acting.

It can be stated that the participating GPs took the opportunity in this survey to state their opinion and let their voices be heard. The perception of the measures implemented about health policy and their impact on primary health care from our results was predominantly negative. The discrepancy between the perceived subjective importance of the GP profession and the consideration granted from the German government and political institutions was lamented as profound. Furthermore, it can be stated that the GPs perceive their work and role as relevant during the COVID-19 pandemic. The government’s interference caused the pandemic to have an even stronger impact on primary health care then the pandemic itself. Accordingly, it can be assumed that it is not exclusively the lack of coordination of the pandemic that is highlighted, but rather that it is the lack of emphasis on the importance of German GPs that is of main concern, as it results in a lack of clarity about the latter’s position and significance in this crisis. The dissatisfaction with political communication and inadequate cooperation is furthermore underpinned by concerns about the working capabilities of the practices and the practice teams themselves.

However, the German part of the PRICOV-19 study wanted to tie up on this, to give the German GPs a voice, and draw them into the centre of attention with link to their professional understanding in such a crisis. The German GPs described a neglect of general practice in the fight against the pandemic, as they received little attention and felt excluded from social as well as health policy appreciation. On the one hand, they are expected to maintain primary health care in the pandemic through specifications and adherence to health policy measures and should act as the trusted representative to their patients in the implementation of the vaccination strategy. On the other hand, GPs felt like they received little consideration and recognition in the German health care system during the pandemic. An automatic dissatisfaction on the part of GPs arose from the lack of recognising that the pursuit of their profession, in its traditional function, sees them fulfilling an obligation to the common good of society, which is especially valuable in this pandemic. Thus, the PRICOV-19 study does not only analyse the organisational and structural problems in the German health care system during the pandemic in primary health care, but also professional political interests also came to light and were openly addressed and critically discussed by the GPs of our survey.

In brief, the GPs were tasked with balancing the values of their profession with the demands of policy within the health care system. GP action is subject to policy requirements in that all measures to contain the COVID-19 pandemic must be prioritised, implemented, and adhered to. As a result of health policy measures, the basis of the legitimacy of GPs’ professional actions is changed and GPs took on roles that were bound by instructions from above. In order to overcome this tension, an active interaction and communication between the primary health care sector and the health policy makers is needed. From the results, it can be stated that a stronger consideration and inclusion of the expertise of GPs in crisis and emergency situations is indispensable. GPs need to be systematically involved in the advisory and feedback structures for policy makers and authorities, and the practical knowledge and existing strength of the ambulatory sector needs to be utilised. An adequate and supportive infrastructure must also be ensured so that GPs can act efficiently [19]. Regular and continuous exchange between primary health care and policy sector is essential for a well-managed pandemic control and prevention as well as for quality assurance in the primary health care sector not only during but also beyond such a pandemic.

### Limitations and strengths

Limitations of this study can be seen in the fact that the participating GPs only represent a small cross section of primary health care professionals that have been impacted by the pandemic. Moreover, as the questionnaire was quite extensive and there was no monetary incentive to participate, it is very likely that GPs who wanted to "make their voices heard" were more likely to participate, which could explain the rather high number of responses. At the same time, we assume that it was probably mainly GPs that actually fared badly in the COVID-19 pandemic who participated in and used this survey as a speaking platform to the outside world.

However, strengths include the way the open text fields offer the possibility for an explicit description of perceived reality. The framework of the online questionnaire and the anonymity provided the space needed to communicate these opinions and the subsequent criticism. Furthermore, there is no limitation to what the answer is allowed to contain, which makes the responses diverse and spontaneous, capturing what the participant already had in mind while being confronted with the topic throughout the questionnaire. Although the structure and the scope of the forgoing questionnaire possibly impacted the content of the open-text answers, they nevertheless provide the opportunity to see which topics GPs wanted to further comment on.

## Conclusion

Overall, the responses received in this analysis reflect a general feeling of exhaustion, disappointment, and frustration by the German GPs with the measures taken by the political authorities during the second COVID-19 wave in February 2021. The health policies were perceived to be even more burdensome on the health system and more detrimental to the provision of quality primary health care than the COVID-19 virus itself. A lack of attention to the GPs with ever increasing responsibility can be seen: due to health policy guidelines, they cannot live out their role in the sense of their GP profession. But precisely the perception and self-image of protecting the common good of society, which this profession stands for, shows that GPs are not just doing “a” job, but acting in the spirit of their medical profession.

In conclusion, this analysis suggests that decisions concerning GPs and their practices should not be made without them–as it is the seamless implementation of these measures that ensures a functioning primary health care system even in times of such a crisis. Our aim was to give participants the opportunity to verbalise what else they had to say. The high number of answers given demonstrated the need to provide an oversight of what the German GPs had at heart.

## Supporting information

S1 File Analysis: Open text field—Categories (English). (PDF)

S2 File Analyse: Offenes Textfeld—Kategorien (German). (PDF)

S3 File Data export. (PDF)

S4 File Data set. (SAV)

S5 File Syntax. (SPS)
