# Supplementary material for: “What else to say?”–Primary health care in times of COVID-19 from the perspective of German general practitioners: An exploratory analysis of the open text field in the PRICOV-19 study
Source: PLoS One. 2023 Mar 17;18(3):e0282504. doi: 10.1371/journal.pone.0282504 (PMC10022805; doi:10.1371/journal.pone.0282504)
Supplement: S1 File — (PDF) [file pone.0282504.s001.pdf]

Analysis: Open text field - Categories (English)

Content

1. Policies and the health system 2

1.1. HA is not given enough prioritisation/protection/support by policy (***no prio***) 2

1.2. Politics /Media Influence on the health system (***policies***) 2

1.3. Influence ASIP (***ASIP***) 3

1.4. Flood of information/changes overwhelming (***info***) 3

1.5. Support (through politics) has improved (***Support***) 3

1.6. Discrepancy policy <-> experiences of the (young) population (***discrepancy***) 4

1.7. Communication politics <-> population (***communication)*** 4

1.8. Quality of care has improved as a result of the pandemic (***quality***) 4

2. Role/Self-perception 4

2.1. Own role as GP:in in the pandemic (***role***) 4

2.2. Self-image (***perception***) 4

3. Cooperation 5

3.1. Cooperation between politics and the medical profession (***PoÄrz***) 5

3.2. Cooperation between Politics and Science (***PoWi***) 5

3.3. Cooperation with other GPs (***GPs***) 5

3.4. Criticism of other HA (***criticism of colleagues***) 5

4. Concerning the practice 5

4.1. Infrastructural adjustments (***Infrastruc***) 5

4.2. Organisation of vaccinations (***vaccination***) 6

4.3. Financial aspects (***finance***) 6

5. Concerning the individual 6

5.1. Change in relation to private life(***Private***) 6

5.2. Mental stress (***Psych***) 6

6. Others 6

7. Index 8

1. Policies and the health system

1.1. HA is not given enough prioritisation/protection/support by policy (*no prio*)

	Statement
T1	At the beginning of the pandemic, I found the work very stressful due to the completely unfamiliar situation despite fewer patients. This has improved over the course of the year with the development of a certain routine private life suffers much more than my professional life from the lockdown. I very much wish for an early vaccination and find it extremely strange that GPs were not included in the first priority level by the politicians. We are only vaccinated earlier so that we don't infect the nursing home patients, but not so that we don't get sick. One can probably get along quite well without us family doctors?!? This aspect makes me sad at the end of my professional career.
T2	The greatest burden is that now that vaccination is available, GPs who care for homes were not extensively prioritised.
T3	The question about patient population (number of patients), but 'NOT note number' is difficult for me as a contract physician to understand; the patient number of our electronic file is at 14500. We care for an apallic respiratory facility (max. 34 beds), a gerontopsychiatric home (not always geronto..... but unfortunately also younger patients; and always palliative patients) and a residential facility for the disabled with three units - in this respect, we are probably not a particularly typical practice; and therefore felt especially let down during the pandemic and as second-class doctors or practice team!
T4	The role of GPs is not strong enough. We have taken care of 90% of covid patients and should not even get vaccination in the first vaccination group. this is very sad and frustrating. To put yourself hejden day in Lebensgefagr and be punished for it with late vaccination is a disaster. Daily changes on the part of the associations of statutory health insurance physicians could hardly be implemented at this pace, especially with the teaching of the staff.
T5	A great relief for all practice staff members would be an early and speedy vaccination !
T6	It's good that they are doing this. Hopefully the politicians will wake up. However, I don't have much hope in this regard. Family medicine has always been neglected, unappreciated and completely underpaid (at least in the KV district of Hamburg). The pandemic and the fight against it will not change anything, on the contrary.
T7	Practice takeover since 2021, therefore partial answers with reference to existing processes required. Little preparation/support from official bodies (KV, ÄK, HZV) for the difficult situation of setting up a business under the influence of the pandemic, especially in rural areas with considerable structural deficits.
T8	Why were GPs and team not in Group 1 for Corona vaccinations from the beginning. We were a hotspot in March 2020 and have not recovered yet. The practice is in danger of falling apart! 3 full time staff have left! So far we have always been a great team and have existed as a large GP practice for 34 years.... At the moment I don't know how to go on.
T9	Another important question is whether one has felt sufficiently informed and supported by the public health system, by the KV, by the local administration during this time. This is not the case for me: I simply do not know any reasonable answers to most of my patients' questions.

1.2. Politics /Media Influence on the health system (*policies*)

	Statement
T10	The chaos caused by politics and the continuous changes in the guidelines have influenced the daily handling of patients and the practice more than the COVID 19 pandemic. The helplessness of politics that still exists today has more negative influence on the health system than the COVID 19 pandemic. Yours sincerely from the countryside
T11	The political coordination is an absolute disaster, no line, no corresponding competence, everything is just confused and everyone does something different. It's all about the next election. There is no reliable guideline for anyone
T12	I care for, among others, 80 opiate substitutes, this group had to suffer a lot under the lockdown, because support systems closed and homeless people no longer had shelters and food was no longer given out, which I found degrading.
T13	Unfortunately, more and more authorities from outside the profession are encroaching on our professional image and patronising us. Health insurance companies are increasingly trying to denounce GPs and push patients away from doctors.

1.3. Influence ASIP[i] (*ASIP*)

	Statement
T14	The worst thing is the KV with thousands of regulations concerning the billing and transfer of smears, extremely complicated.
T15	The ongoing KV notifications and many changes in the Corona procedure and the fight for disinfectants etc. we're annoying. There was not as much time as before for other further training. The patients are increasingly irritated. The nurses also have a second job, home-schooling, which eats up additional energy. Nevertheless, life is fun - even in a team!
T16	The role of GPs is not strong enough. We have cared for 90% of Covid patients and should not even get vaccinated in the first vaccination group. this is very sad and frustrating. To put yourself hejden day in Lebensgefagr and be punished for it with late vaccination is a disaster. Daily changes on the part of the associations of statutory health insurance physicians could hardly be implemented at this pace, especially with the teaching of the staff.
T17	I took over the practice in April 2020 and do not feel supported by the KV. Didn't benefit from the KV's rescue package, even though the number of patients dropped like all other practices. They don't calculate my rescue fund with 917-1000 patients like my predecessor had, but with 719 patients, which we will never fall below. There were hardly any patients leaving the practice because the area was underserved. However, due to modernisation and increased prices for hygiene, I have more expenses than my predecessor. The first payment on account was 7000 € instead of 14000 € because the KV Berlin boards had decided so for newly taken over practices. Emergency day care did not work either and had to be paid privately.

1.4. Flood of information/changes overwhelming (*info*)

	Statement
T18	The information of politics via the media and what I can implement in my practice did not agree at any time. Decisions are made that I cannot implement because material or prerequisites are missing. So now it is happening again with the rapid tests in my practice. How am I supposed to be able to offer a quick test to anyone who wants it, when I am paying for it, don't have that much quick test, have an infection consultation and treat sick people. It is not my job to provide healthy people with rapid tests - e.g. the almost weekly change of billing modalities regarding COVID in autumn 2020 (teachers, travel returnees, contact persons etc.) was an imposition to say the least and almost impossible to implement. The flood of weekly, even daily information and the nonsense spread in the press regarding COVID weighs more on my mind than the treatment and handling of patients.
T19	I found the incredible flood of information with the many, very rapid changes (to which we as panel doctors were exposed last year and to which we always had to react at lightning speed) very stressful last year!
T20	With calm and confidence we will get through the pandemic! The flood of information from different agencies is very exhausting! One can easily lose the overview and attention !
T21	The daily - weekly changing requirements for billing, tests and communication with the health authorities are very stressful. This has increased by at least 1/3 and ties up a lot of energy and time, which is then lacking for direct patient contact.
T22	It's not so much patients or illnesses that stress us out, but rather the constant change of regulations, changed orders, guidelines, etc. Often you find out on Friday afternoon what will change on Monday, and then on Tuesday that it should be done differently... (e.g. PCR/POC tests!).
T23	Too much to read, decrees too much and too late
T36	there is too little information about outpatient COVID therapy

1.5. Support (through politics) has improved (*Support*)

	Statement
T24	At the beginning of the pandemic, government support, e.g. through protective clothing, was insufficient. This has improved significantly.
T25	Support during the pandemic came from the GP association with practical tips

1.6. Discrepancy policy <-> experiences of the (young) population (*discrepancy*)

	**Statement**
T26	As GPs, we experience a discrepancy between statements, figures, political action and experience on the front line.the psychological/physical, social damage, especially to the young, is frightening, the measures disproportionate. More common sense and the courage to move forward, optimism, less fear-mongering and mobilising all forces for vaccinations would be my wish.

1.7. Communication politics <-> population (*communication)*

	**Statement**
T27	Pitiful is the ignorance of the people even after one year of pandemic. The state as educator has failed (exercise, vitamins, mask science). Communicationgets a straight five.

1.8. Quality of care has improved as a result of the pandemic (*quality*)

	**Statement**
T28	Covid was the opportunity for many organisational and structural improvements in outpatient care, which we would like to pursue further in the future. A lethargic care system has been shaken up, has become more focused on its core tasks and has sought digital progress. The quality of health care has gained from the pandemic.

2. Role/Self-perception

2.1. Own role as GP:in in the pandemic (*role*)

	**Statement**
T29	Dealing with the pandemic is part of my job, which means more work and a strong focus on one topic, but also more family time due to the elimination of many appointments in the lockdown, especially in the first lockdown, which I appreciated very much. I am grateful for being able to continue working without being affected by it, compared to other professions. I am doing my part in coping with the pandemic and, if possible, giving our patients support and peace of mind.
T4	The role of GPs is not strong enough. We have taken care of 90% of covid patients and should not even get vaccination in the first vaccination group. this is very sad and frustrating. To put yourself hejden day in Lebensgefagr and be punished for it with late vaccination is a disaster. Daily changes on the part of the associations of statutory health insurance physicians could hardly be implemented at this pace, especially with the teaching of the staff.

2.2. Self-image (*perception*)

	**Statement**
T31	I find the Corona time unmasking for the social attitude of the individual team members. I am very grateful for the willingness of most of them to roll up their sleeves and lend a hand. In the end, we have moved together as a team.
T32	I see the pandemic as a great danger and challenge. But I also see it as a fundamental medical task to face this challenge.
T33	My big wish for the politicians is that doctors should be paid for their telephone calls even after the pandemic, because making free calls for 4 hours a day is unworthy of our profession. The telephone calls can reduce the possibility of infection in the practices and the patients are still well cared for. Corona per se has not increased my workload (the additional bureaucratic work and the reduced number of patients balance each other out).
T34	Insufficient appreciation in financial and political terms Role and tasks of GPs , lack of co-participation Vaccination Covid-19
T35	We would like to see MFAs receive a covid surcharge, as the GP association has been demanding for a long time. And on the other hand, it would be motivating if politicians alsovalued the services of the outpatient care level more, especially GP care.

3. Cooperation

3.1. Cooperation between politics and the medical profession (*PoÄrz*)

	Statement
T37	It would be nice if the responsible bodies were not so over-bureaucratised and if, for example, accounting etc. could be done SIMPLY instead of 8 times.
T38	It is about time that GPs are noticed by politics and administration. We could have been involved in vaccination long ago. But there is not even information on how this will happen when the vaccine wave rolls in. The role of the Medical Association is pathetic. So is the communication with us doctors.

3.2. Cooperation between Politics and Science (*PoWi*)

	Statement
T39	The federal and state governments should not follow the advice of individual experts of their own choosing, but should listen to a predetermined panel of various experts. The Basic Law and parliamentary democracy should not be so easily overridden. The Minister of Health should be compelled to include and consult medical experts from different disciplines in his ministry.

3.3. Cooperation with other GPs (*GPs*)

	Statement
T40	Since COVID, our practice has shared the infection consultation hours with the neighbouring practice, so that we only had infection consultation hours 3 afternoons a week and could also find time for the vaccinations and check-ups.

3.4. Criticism of other HA (*criticism of colleagues*)

	Statement
T41	The question of whether colleagues have prepared themselves for the pandemic is missing. We are not in the school where the government has to do everything for us. You should also be able to read the signs of the times yourself. When I appeared on whatsapp with protective clothing Faceshield and mask on 23.2. 2020 in whatsapp I got a shitstorm. On 1.3. we had masks, partitions and protective clothing such as triage and extra consultation hours for patients with infections. Many colleagues were still dreaming at that time. None of my staff and family have ever been sick, we are working more than before but we are proud to have supported our community and surrounding area as a test practice and care practice. We have 1/3 more patients due to coronary disease but this will be remedied. We have never had a shortage of masks, disinfection material or protective clothing for the practice, our staff or our families.

4. Concerning the practice

4.1. Infrastructural adjustments (*Infrastruc*)

	Statement
T42	Your questionnaire did not include/address the practices that have themselves made a separation of infected and 'normal' potentially non-infected patients through reconstruction measures. We have set up a container outside, in front of the practice, and do our own swabs as well as diagnostics and therapy for all infected patients - in the container. This protects the 'normal' patients and gives them confidence to go to the doctor.
T43	The practice could only be organised so well because we have a separate seminar room, which is located opposite in another building, and can be used for infection consultations.
T44	We are a corona smear practice. We have set up tents in front of the practice to do triads and smears.separation between infection consultation and other illnesses.
T45	Strict separation of patients: Infectious disease consultation at extra times in a room used only for this purpose outside the normal practice.
T46	We have permanently rebuilt the registration desk. We have installed hygiene stations at three entrances. We moved into our newly built practice 2 years ago, which we planned for this scenario at the time of swine flu, so we are coping very well!

T47	We are now better organised in the practice, the cohesion is better and the patients cooperate well. We have the advantage of having two entrances I see all the Covid patients alone in a separate room, only I have contact with them. The MFAs are completely protected. Covid 19 has changed a lot of things, as bad as they are, for the better. We are also already vaccinated, as we look after three AHs and hold infection consultations. We are already trying hard to prepare for the Corona vaccinations and have extended the consultation hours from April 2021 Overall, the burden for a single doctor's practice is very high because the quarantine hovered over us until the vaccination, which was the worst. It will be fine

4.2. Organisation of vaccinations (*vaccination*)

	**Statement**
T48	Besides Covid, the issue of vaccinations was problematic. The flu vaccination was strongly desired by our patients, vaccine came too little too late. Pneumococcal 23 vaccination has not been available for us for over 1 year. I fear that the Covid vaccinations will be delegated back to the GP practices too soon by the politicians after the appropriate vaccines have been approved, and that we will once again have to explain ourselves to our patients, through no fault of our own, due to a lack of sufficient vaccine quantities ... let's see what happens...
T49	Annoyance about the very poor organisation of vaccinations in general and especially for elderly patients cared for at home.
T50	Please no 3rd wave - vaccination for all without prioritisation as soon as possible by GPs

4.3. Financial aspects (*finance*)

	**Statement**
T51	I am worried about my finances. Rising staff costs (MFA collective agreement) and falling income (patient numbers) are currently causing a new financial 'imbalance'.
T52	In phases of lockdown, patient volumes in GP practices decline significantly with considerable financial and economic risks for the practices.
T53	My big wish for the politicians is that doctors should be paid for their telephone calls even after the pandemic, because making free calls for 4 hours a day is unworthy of our profession. The telephone calls can reduce the possibility of infection in the practices and the patients are still well cared for. Corona per se has not increased my workload (the additional bureaucratic work and the reduced number of patients balance each other out).

5. Concerning the individual

5.1. Change in relation to private life(*Private*)

	**Statement**
T54	The ongoing KV notifications and many changes in the Corona procedure and the fight for disinfectants etc. were annoying. There was not as much time as before for other further training. The patients are increasingly irritated. The nurses also have a second job, homeschooling, which eats up additional energy. Nevertheless, life is fun - even in a team!

5.2. Mental stress (*Psych*)

	**Statement**
T55	I have been thinking for a short time - since my colleague is also in burn-out - about how to continue. Due to additional burdens (TI/ telematics implementations, more and more bureaucracy and too many changing requirements) I am already thinking about GIVING UP the practice!

6. Others

	**Statement**

T56	What doesn't really come out in the questionnaire is the disruptive aspect of the pandemic with regard to any routine. It would be an exciting field to question how crises (pandemic, climate, etc.) affect our individualised patient care now and in the future and with which tools we can better integrate this.
T57	There is a lack of differentiation between the role of'government' and medical bodies
T58	It would be interesting to (partially) repeat the survey if Corona vaccinations had to be carried out even more frequently in vaccination centres or in GP practices in addition to normal practice operations. The organisational and time-related Problems would then increase massively!
T59	Answer ' don't know' to the questions about painting centres, as we ourselves carry out testing in large numbers separately from normal practical operations.
T60	there could be a little less question! i would very much like to receive the results of this evaluation as well
T61	Please send me the results of the survey.
T62	Would like to be informed about the results of the study.
T63	I would be interested to know how this survey turned out.
T64	The question of whether patients were treated later because of the Corona Triage is unclear to me. Are one to 3 hours to max. 1 day later relevant later? I have understood and answered it in this way, although I do not consider it relevant and there has been no disadvantage for any patient.
T65	I run a private rural medical practice and am not comparable to an MVZ. That is a big difference. I could not find any differentiated questions on this.
T66	Some of the questions are too narrow. It shows a university distance to the practice... despite the fact that the survey was conducted by one of our departments...?
T67	Some questions were incomprehensible
T68	How should a figure be given for the patient base? All in the database? hardly - does not reflect the patient volume. A mock number would be better.
T69	some questions should have been answered with 'does not happen'. so i just wrote what i would have done if it had happened.
T70	... the questionnaire was a little long, in part imprecisely formulated, and sometimes the meaning of the question was not clear.
T71	I took the question with the review literally
T72	the survey has no connection with corona at all, but aims at a workplace survey/analysis regarding general medicine/migration
T73	The question about the number of patients is completely unclear to me, we have about 2,300 to 2,400 notes per quarter. That's why I filled it in with 0000.
T74	Please avoid abbreviations in your cover letter, e.g. PSA! You cannot always assume that everyone will understand your abbreviations.
T75	Interview was a bit too long, I thought about stopping several times
T76	Well designed study
T77	Good that you shed light on these aspects of COVID
T78	Thank you - interesting questions for me to reflect on.
T79	In my opinion, the infection event does not correspond to the communicated severity
T80	I am happy to be able to work in my country - when I see the situation in other countries ...
T81	I assume that the legal procedures are actually based on KV guidelines, i.e. billing figures and methods. After all, I am self-employed and can do everything the way I want.
T82	separate doctor's case I found a suggestion, whereby we disinfect all contaminated equipment (stethoscope etc.).
T83	We don't have online consultation hours, but we do have appointment-only consultation hours. Even before corona .... I tried to transfer this! Best support came from degam benefits
T84	We have been a Covid focal point practice for 1 year. I am the head infectious disease doctor there.

7. Index

Changes 1, 2, 3, 5, 9

Occupation 5

Influence 1, 2, 3	Vaccination 1, 2, 3, 5, 7
Decisions 3	Associations of SHI-accredited physicians 2, 3, 5
Experts 6	Colleagues 6
Health care 4	Communication 1, 3, 4, 6
GPs 2, 3, 5, 7	Health insurance funds 3
GP practice 2, 7	Health insurance (KV)1, 2, 3, 5, 9, 11
Challenge 5	Lockdown 2, 5
Hygiene stations 7	Shortage 6

[i] Association of Statutory Insurance Physicians
