# Supplementary material for: “What else to say?”–Primary health care in times of COVID-19 from the perspective of German general practitioners: An exploratory analysis of the open text field in the PRICOV-19 study
Source: PLoS One. 2023 Mar 17;18(3):e0282504. doi: 10.1371/journal.pone.0282504 (PMC10022805; doi:10.1371/journal.pone.0282504)
Supplement: S2 File — (PDF) [file pone.0282504.s002.pdf]

Analyse: Offenes Textfeld – Kategorien (German)

Inhalt

1. Politik und Gesundheitssystem 2

1.1. HA wird von der Politik nicht genügend Priorisierung/Schutz/Unterstützung zugewiesen (***no Prio***) 2

1.2. Politik/Medien Einfluss auf das Gesundheitssystem (***Politik***) 2

1.3. Einfluss KV (***KV***) 3

1.4. Flut an Informationen/Änderungen überfordernd (***Info***) 3

1.5. Unterstützung (durch Politik) hat sich gebessert (***Support***) 3

1.6. Diskrepanz Politik <-> Erfahrungen der (jungen) Bevölkerung (Diskrepanz) 3

1.7. Kommunikation Politik <-> Bevölkerung (***Kommunikation***) 4

1.8. Qualität der Versorgung hat durch die Pandemie dazu gewonnen (***Quali***) 4

2. Selbstbild/Rolle 5

2.1. Eigene Rolle als Hausarzt:in in der Pandemie (***Rolle***) 5

2.2. Selbstbild (***Bild***) 5

3. Zusammenarbeit 5

3.1. Zusammenarbeit Politik und Ärzteschaft (***PoÄrz***) 5

3.2. Zusammenarbeit Politik und Wissenschaft (***PoWi***) 5

3.3. Zusammenarbeit mit anderen HA (***ÄrzÄrz***) 6

3.4. Kritik an anderen HA (***Kritik Kolleg:innen***) 6

4. Die Praxis betreffend 6

4.1. Infrastrukturelle Anpassungen (***Infrastruk***) 6

4.2. Organisation der Impfungen (***Impfung***) 6

4.3. Finanzielle Aspekte (***Finanz***) 7

5. Das Individuum betreffend 7

5.1. Veränderung im Bezug auf das Privatleben (***Privat***) 7

5.2. Psychische Belastung (***Psych***) 7

6. Sonstiges 7

7. Index 8

1. Politik und Gesundheitssystem

1.1. HA wird von der Politik nicht genügend Priorisierung/Schutz/Unterstützung zugewiesen (*no Prio*)

**Aussage**
Am Anfang der Pandemie habe ich die Arbeit durch die völlig ungewohnte Situation trotz weniger Patienten als sehr belastend empfunden. Das hat sich im Laufe des Jahres mit Entwicklung einer gewissen Routine gebessert. Das Privatleben leidet deutlich mehr als das Berufsleben unter dem Lockdown. Ich wünsche mir sehr eine baldige Impfung und finde es äußerst seltsam, dass Hausärzte von der Politik nicht in die erste Prioritätsstufe aufgenommen wurden. Wir werden nur deshalb früher geimpft, damit wir die Altenheimpatienten nicht anstecken, aber nicht, damit wir nicht krank werden. Man kann wohl auch ganz gut ohne uns Hausärzte auskommen?!? Dieser Aspekt macht mich am Ende meiner Berufslaufbahn dann schon traurig
Am stärksten belastet, das jetzt wo eine Impfung verfügbar ist, die Hausärzte, die Heime versorgen, nicht flächendeckend priorisiert werden
Die Frage nach der Patientenpopulation (Zahl der Patienten), aber 'NICHT-Scheinzahl' ist für mich als Vertragsarzt schwer zu verstehen; die Patientennummer unserer elektronischen Kartei steht bei 14500. Wir versorgen eine Apalliker-Beatmungseinrichtung (max. 34 Betten), ein gerontopsychiatrisches Heim (nicht immer geronto..., leider auch immer wieder Jüngere; und immer palliative Patienten) und eine Behinderten-Wohneinrichtung mit drei Einheiten - insofern dürften wir eine nicht besonders typische Praxis sein; und fühlten uns in der Pandemie deswegen auch und gerade besonders im Stich gelassen und als Ärzte bzw. Praxisteam zweiter Klasse!
Die Rolle der Hausärzte ist nicht stark genug. Wir haben 90% der Covid-Patienten versorgt und sollten noch nicht einmal in der ersten Impfgruppe eine Impfung bekommen. das ist sehr traurig und frustrierend. Sich hejden Tag in Lebensgefagr begeben und dafür mit Spätimpfung abgestraft zu werden, ist eine Katastrophe. Tägliche Änderungen seitens der Kassenärztlichen Vereinigungen waren in dem Tempe insbesondere mit Teaching der Mitarbietriennen kaum umzusetzen.
Eine große Entlastung für alle Mitarbeitenden in unserer Praxis wäre eine baldige Impfung!
Gut, dass sie das machen. Hoffentlich wacht die Politik dann auf. Viel Hoffnung habe ich diesbezüglich allerdings nicht. Die Hausarztmedizin wird seit eh und jeh stark vernachlässigt, nicht gewürdigt und völlig unterbezahlt (jedenfalls im KV-Bezirk Hamburg). Daran wird auch die Pandemie und deren Bekämpfung nichts ändern, im Gegenteil.
Praxisübernahme seit 2021, daher teilweise Antworten unter Bezugnahme auf bestehende Prozesse erforderlich. Durch die offiziellen Stellen (KV, ÄK, HZV) wenig Vorbereitung/Unterstützung für die erschwerte Situation einer Unternehmensgründung unter Pandemieeinflüssen, insbesondere im ländlichen Raum mit erheblichen Strukturdefiziten.
Warum waren Hausärzte und Team nicht von Beginn an in Gruppe 1 für die Corona-Impfungen. Wir waren im März 2020 ein Hotspot und haben uns bis jetzt nicht erholt. Die Praxis droht zu zerfallen! 3 Vollzeitkräfte sind gegangen! Bisher waren wir immer ein tolles Team und bestehen seit 34 Jahren als große Hausarztpraxis .... Zur Zeit weiß ich nicht, wie es weitergehen wird.
Wichtig wäre noch die Frage, ob man sich in dieser Zeit genug informiert und unterstützt gefühlt hat, vom öffentlichen Gesundheitswesen, von der KV, von der örtlichen Verwaltung. Das ist bei mir eben gar nicht der Fall: ich weiß auf die meisten Fragen meiner PatientInnen einfach keine vernünftige Antwort.

1.2. Politik/Medien Einfluss auf das Gesundheitssystem (*Politik*)

**Aussage**
Das durch die Politik hervorgerufene Chaos und die andauernden Änderungen der Vorgaben haben den täglichen Umgang mit den Patienten und dem Praxisgeschehen mehr beeinflusst als die COVID 19 Pandemie. Die auch heute noch bestehende Hilflosigkeit der Politik nimmt mehr negativen Einfluss auf das Gesundheitssystem als die COVID 19 Pandemie. Mit freundlichen Grüßen vom Lande
Die politische Koordination ist eine absolute Katastrophe, keine Linie, keine entsprechende Kompetenz, alles nur wirr und jeder macht was anderes. Es geht um die nächste Wahl. Es existiert für niemanden ein verlässlicher Leitfaden
ich versorge ,unter anderem, 80 Opiatsubstituierte, diese Gruppe hatte unter dem lockdown sehr zu leiden, da Hilfesysteme schlossen und Obdachlose keine Unterkünfte mehr hatten und kein Essen mehr ausgegeben wurde, das fand ich menschenunwürdig
Leider drängen sich immer mehr fachfremde Instanzen in unser Berufsbild und bevormunden. Krankenkassen versuchen zunehmend insbesondere Hausärzte zu denunzieren und Patienten vom Arzt abzudrängen.

1.3. Einfluss KV (*KV*)

Aussage
das schlimmste ist die KV mit tausenden Regelungen bzgl. der Abrechnung und Überweisung der Abstriche, extrem kompliziert
Die laufenden KV Mitteilungen und vielen Änderungen im Corona Procedere und der Kampf um Desinfektionsmittel etc nervten. Dabei war nicht mehr soviel Zeit wie vorher für andere Weiterbildungen. Die Patienten sind zunehmend gereizter. Die Schwestern haben noch den Zweitjob Homeschooling, der zusätzlich Energie frisst. Das Leben macht trotzdem Spaß- auch im Team!
Die rolle der Hausärzte ist nicht stark genug. Wir haben 90% der Covid-Patienten versorgt und sollten noch nicht einmal in der ersten Impfgruppe eine Impfung bekommen. das ist sehr traurig und frustrierend. Sich hejden Tag in Lebensgefagr begeben und dafür mit Spätimpfung abgestraft zu werden, ist eine Katastrophe. Tägliche Änderungen seitens der Kassenärztlichen Vereinigungen waren in dem Tempe insbesondere mit Teaching der Mitarbietriennen kaum umzusetzen.
Habe die Praxis im April 2020 übernommen und fühle mich durch KV nicht unterstützt. Profitierte nicht vom Rettungsschirm der KV obwohl Patienteneinbruch wie alle Praxen. Sie berechnen meinen Rettungsschirm nicht mit 917 -1000 wie Vorgängerin hatte sondern 719 Patienten, welche wir nie unterschreiten werden. Einen Praxisabgang von Patienten gab es kaum da unterversorgtes Gebiet. Ich habe aber durch die Modernisierung und gestiegenen Preise fürHygiene mehr Ausgaben als meine Abgeberin. Die erste Abschlagszahlung lag bei 7000 € anstelle 14000 weil es die KV Berlin Vorstände so beschlossen hatten für Neu übernommene Praxen. Kita Notbetreuung funktionierte auch nicht und musste privat bezahlt werden.

1.4. Flut an Informationen/Änderungen überfordernd (*Info*)

Aussage
Die Information der Politik über die Medien und das was ich in meiner Praxis umsetzen kann stimmte zu keiner Zeit über ein. Es werden Entscheidungen getroffen, die ich nicht umsetzen kann, weil Material oder die Voraussetzungen fehlen. So jetzt wieder geschehen mit den Schnelltestung in meiner Praxis. Wie soll ich hier jedem einen Schnell Test, der es wünscht anbieten können wo ich zum einen drauf bezahle, so viel schnelltest nicht habe, Infektionssprechstunde habe und kranke behandle. Es ist nicht auf meine Aufgabe gesunde Menschen mit schnelltest zu versorgen -z bsp. Die fast wöchentlich Änderung der Abrechnungsmodalitäten bezüglich COVID im Herbst 2020 (Lehrer, Reise Rückkehrer, Kontakt Personen etc.) war gelinde gesagt eine Zumutung und fast nicht umsetzbar. Die Flut an wöchentlichen, ja täglichen Informationen und der Unsinn der in der Presse bezüglich COVID verbreitet wird belastet mich mehr als die Behandlung und der Umgang mit den Patienten.
Ich fand die unglaubliche Informationsflut mit den vielen, sehr schnellen Änderungen (denen wir als Kassenärzte im letzten Jahr ausgesetzt waren und auf die wir immer blitzschnell reagieren mussten) im letzten Jahr sehr belastend!
mit Ruhe und Zuversicht werden wir die Pandemie überstehen! DIe Flut an Informationen durch verschiedene Stellen ist sehr anstrengend! Kann man leicht den Überblick und die Aufmerksamkeit verlieren !
Sehr belastend sind die täglich - wöchentlich sich ändernden Vorgaben für Abrechnung, Testungen , sowie die Kommunikation mit dem Gesundheitsamt. Dies hat um mind.1/3 zugenommen und bindet sehr viel Kraft und Zeit, die dann für den direkten Patientenkontakt fehlt
Weniger Pat. oder Krankheiten stressen uns, vielmehr der ständige Wechesel von Bestimmungen, geänderte Anordnungen, Vorgaben etc. Oft erfährt man am Fr. Nachmittag, was sich am Mo. ändern wird, und am Di dann, dass es doch anders gemacht werden sollte...(Bsp. PCR/POC-Tests!)
Zuviel zu lesen, Erlasse zu viel und zu spät

1.5. Unterstützung (durch Politik) hat sich gebessert (*Support*)

Aussage
Am Anfang der Pandemie war die Unterstützung durch die Regierung z.B. durch Schutzkleidung nicht ausreichend. Dies hat sich deutlich gebessert.
Unterstützung während der Pandemie kam vom Hausärzteverband mit praktischen Tipps

1.6. Diskrepanz Politik <-> Erfahrungen der (jungen) Bevölkerung (Diskrepanz)

Aussage

Als HÄ erleben wir eine Diskrepanz zwischen Aussagen,Zahlen,Handeln der Politik und Erleben an der Front.die psych/physisch., sozial.Schäden va.der Jungen sind erschreckend,die Massnahmen unverhältnismäßig. Stat.zahlen und Realität Klaffen auseinander.mehr ges.menschenverstand und mut zum vorangehen,Optimismus ,weniger Angstmache und alle Kräfte für Impfungen mobilisieren wäre mein wunsch

1.7. Kommunikation Politik <-> Bevölkerung (*Kommunikation*)

**Aussage**
Erbarmungswürdig ist die Unwissenheit der Menschen auch nach einem Jahr Pandemie. Der Staat als Aufklärer hat versagt (Bewegung, Vitamine, Maskenkunde). Die Kommunikation erhält eine glatte fünf.

1.8. Qualität der Versorgung hat durch die Pandemie dazu gewonnen (*Quali*)

**Aussage**
Covid war die Chance für viele organisatorische und strukturelle Verbesserungen in der ambulanten Versorgung, die wir künftig weiter verfolgen möchten. Ein lethargiegefährtes Versorgungssystem wurde aufgerüttelt, hat sich mehr auf seine Kernaufgaben besonnen und sich um digitalen Fortschritt bemüht. Die Qualität der Gesundheitsversorgung hat durch die Pandemie gewonnen.

2. Selbstbild/Rolle

2.1. Eigene Rolle als Hausarzt:in in der Pandemie (*Rolle*)

Aussage
Der Umgang mit der Pandemie gehört zu meinem Beruf, das bedeutet Mehrarbeit und starke Fokussierung auf ein Thema, durch den Wegfall vieler Termine im Lockdown allerdings auch zu mehr Familienzeit, insbesondere im 1. Lockdown, die ich sehr geschätzt habe. Ich bin dankbar dafür, recht unbeeinträctigt weiterarbeiten zu können im vergleich zu anderen Berufsgruppen. Ich leiste meinen Anteil an der Bewältigung der Pandemie und gebe unseren Patienten wenn möglich Halt und Ruhe.
Die rolle der Hausärzte ist nicht stark genug. Wir haben 90% der Covid-Patienten versorgt und sollten noch nicht einmal in der ersten Impfgruppe eine Impfung bekommen. das ist sehr traurig und frustrierend. Sich hejden Tag in Lebensgefagr begeben und dafür mit Spätimpfung abgestraft zu werden, ist eine Katastrophe. Tägliche Änderungen seitens der Kassenärztlichen Vereinigungen waren in dem Tempe insbesondere mit Teaching der Mitarbietriennen kaum umzusetzen.

2.2. Selbstbild (*Bild*)

Aussage
ich finde die Coroanzeit demaskierend für die soziale Einstellung der einzelnen Team-Mitglieder. Über die Bereitschaft der allermeisten die Ärmel hoch zu krämpeln und anzupacken bin ich sehr dankbar. Letzendlich sind wir als Team zusammen gerückt
Ich sehe die Pandemie als große Gefahr und Herausforderung. Aber ich sehe es auch als grundsätzliche ärztlich Aufgabe sich dieser Herausforderung zu stellen
Mein großer Wunsch an die Politik: ärztliche Telefonate auch nach der Pandemie zu vergüten, denn 4h/d gratis zu telefonieren, ist unseres Berufstandes unwürdig. Durch die Telefonate können Ansteckungsmöglichkeiten in den Praxen dtl. reduziert werden und die Patienten sind trotzdem gut versorgt. Corona per se hat meine Arbeitsbelastung nicht erhöht (der bürokratische Mehraufwand und die verminderte Patientenzahl halten sich die Waage).
Unzureichende Würdigung in finanzieller und politischer Hinsicht Rolle und Aufgaben der Hausärzte, fehlende Mitbeteiligung Impfung Covid-19
Wir würden uns wünschen, daß zum einen MFA's einen Covid-Zuschlag erhalten, so wie es der Hausarztverband schon lange fordert. Und zum anderen wäre es motivierend, wenn auch die Politik die Leistungen der ambulanten Versorgungsebene und hier insbesondere die hausärztliche Versorgung mehr wertgeschätzt würde.

3. Zusammenarbeit

3.1. Zusammenarbeit Politik und Ärzteschaft (*PoÄrz*)

Aussage
es gibt zu wenige Informationen über die ambulante COVID-Therapie
Es wäre schön, wenn die zuständigen Stellen nicht so überbürokratisiert wären und zB Abrechnung etc EINFACH statt zT 8-fach erledigt werden könnte.
Es wird zeit, Dass hHausärzte überhaupt wahrgenommen werden von Politik und Administration. Wir könnten schon längst ins Impfen eingebunden sein. Aber es gibt noch nicht einmal Informationen, wie dies geschehn wird, wenn die Impfstoffwelle anrollt. Die Rolle der Ärztekammer ist erbärmlich. Die Kommunikation mit uns Ärzten auch.

3.2. Zusammenarbeit Politik und Wissenschaft (*PoWi*)

Aussage
Die Bundesregierung und Länderregierungen sollten nicht den Rat einzelner selbstgewählter Experten befolgen, sondern ein vorgegebenes Gremium aus verschiedenen Experten hören. Das Grundgesetz und die parlamentarische Demokratie darf nicht so einfach Außerkraft gesetzt werden können. Der Gesundheitsminister sollte zwangsweise Mediziner aus verschiedenen Sparten in seinem Ministerium enthalten haben und konsultieren müssen

3.3. Zusammenarbeit mit anderen HA (*ÄrzÄrz*)

Aussage
Seit COVID hat sich unsere Praxis mit der Nachbarpraxis die Infektsprechstunde geteilt, sodass wir nur an 3 Nachmittagen in der Woche Infektsprechstunde hatten und auch so Zeit für die Impfungen und Check ups finden konnten.

3.4. Kritik an anderen HA (*Kritik Kolleg:innen*)

Aussage
Es fehlt die Frage ob die Kollegen sich selber auf die Pandemie vorbereitet haben. wir sind nicht in der Schule dass die Regierung alles machen für uns muss. Man sollte auch selbst die Zeichen der Zeit lesen können. Als ich mit Schutzkleidung Faceshield und Maske am 23.2..2020 in whatsapp auftrat erntete ich einen Shitsturm . Am 1.3. hatten wir Masken, Trennwände und Schutzkleidung wie Triage und extra Sprechzeiten für Patienten mit Infekten. Viele Kollegen haben zu dieser Zeit noch geträumt. Keiner meiner Angestellten mit Familie waren je krank, wir arbeiten mehr als vorher aber wir sind stolz als Testpraxis und Versorgungspraxis unsere Gemeinde und Umgebung unterstützt zu haben Wir haben koronabedingt 1/3 mehr Patienten, aber das wird sich wieder geben.Wir hatten nie einen Mangel an Masken, Desinfektionsmaterial oder Schutzkleidung für die Praxis, unser Personal oder unsere Familien

4. Die Praxis betreffend

4.1. Infrastrukturelle Anpassungen (*Infrastruk*)

Aussage
Ihr Fragebogen hat nicht die Praxen eingeschlossen/angesprochen, die selbst durch Umbaumaßnahmen eine Trennung von Infizierten und 'normalen' potentiell nicht Infizierten Patienten vorgenommen hat. Wir haben einen Container aufgebaut draußen, vor der Praxis und machen selber Abstriche sowie Diagnostik und Therapie aller Infizierten - im Container. Das schützt die 'normalen' Patienten und gibt denen auch Zuversicht, zum Arzt gehen zu können
Praxis ließ sich nur so gut organisieren, weil wir über einen separaten Seminarraum verfügen, der gegenüber in einem anderen Gebäude liegt, und zur Infektsprechstunde genutzt werden kann.
Sind Corona-Abstrich-Praxis.Haben Zelte vor der Praxis aufgebaut um dort Triade und Abstriche zu machen.Trennung zwischen Infektsprechstunde und anderen Erkrankungen
Strikte Trennung der Patienten: Infektionssprechstunde zu Extra- Zeiten in nur dafür genutztem Raum außerhalb der normalen Praxis
Wir haben die Anmeldung dauerhaft umgebaut. Wir haben Hygienestationen an drei Eingängen angebracht. Wir haben vor 2 Jahren unsere neu gebaut Praxis bezogen, die wir zu Zeiten der Schweinegrippe für dieses Szenario geplant haben und so bestens zurecht kommen!
Wir sind nun besser in der Praxis organisiert, der Zusammenhalt ist besser und die Patienten machen gut mit. Wir haben den Vorteil zwei Eingänge zu haben Ich untersuche alle Covid Patienten allein in einem seperatem Raum, nur ich habe Kontakt mit diesen. Die MFA sind völlig geschützt. Covid 19 hat vieles, so schlimm das auch ist, zum Guten verändert. Auch sind wir schon geimpft, da wir drei AH betreuen und Infektsprechstunden abhalten. Wir versuchen auch jetzt schon uns sehr auf die Coronaimpfungen vorzubereiten und haben die Sprechstunden ab April erweitert 2021 Insgesamt ist die Belastung für eine Einzelarztpraxis sehr hoch weil die Quarantäne bis zur Impfung über uns schwebte, das war das schlimmste. Es wird schon

4.2. Organisation der Impfungen (*Impfung*)

Aussage
Problematisch war neben Covid das Thema Impfungen. Die Grippeimpfung war seitens unserer Patienten stark erwünscht, Impfstoff kam zu wenig zu spät. Pneumokokken 23 Impfung ist für uns seit über 1 Jahr nicht verfügbar. Ich befürchte, dass die Covid-Impfungen nach entsprechender Zulassung tauglicher Impfstoffe zu früh von der Politik auf die Hausarztpraxen rückdelegiert werden und wir mangels ausreichender Impfstoffmengen wieder unseren Patienten gegenüber, ohne dass wir etwas dafür können, in Erklärungsnot geraten... let's see what happens...
Ärger über die sehr schlechte Organisation der Impfungen generell und speziell für zuhause versorgte alte Pat
Bitte keine 3. Welle - Impfung für Alle ohne Priorisierung so schnell wie möglich durch die Hausärzte

4.3. Finanzielle Aspekte (*Finanz*)

Aussage
Ich mache mir finanzielle Sorgen. Steigende Personalkosten (MFA-Tarifvertrag) und sinkende Einnahmen (Patientenzahlen) verursachen aktuell eine neue finanzielle 'Schieflage'.
In Phasen des Lockdowns ist das Patientenaufkommen in der Hausarztpraxis deutlich rückläufig mit erheblichen finanziellen und wirtschaftlichen Risiken für die Praxen.
Mein großer Wunsch an die Politik: ärztliche Telefonate auch nach der Pandemie zu vergüten, denn 4h/d gratis zu telefonieren, ist unseres Berufstandes unwürdig. Durch die Telefonate können Ansteckungsmöglichkeiten in den Praxen dtl. reduziert werden und die Patienten sind trotzdem gut versorgt. Corona per se hat meine Arbeitsbelastung nicht erhöht (der bürokratische Mehraufwand und die verminderte Patientenzahl halten sich die Waage).

5. Das Individuum betreffend

5.1. Veränderung im Bezug auf das Privatleben (*Privat*)

Aussage
Die laufenden KV Mitteilungen und vielen Änderungen im Corona Procedere und der Kampf um Desinfektionsmittel etc nervten. Dabei war nicht mehr soviel Zeit wie vorher für andere Weiterbildungen. Die Patienten sind zunehmend gereizter. Die Schwestern haben noch den Zweitjob Homeschooling, der zusätzlich Energie frisst. Das Leben macht trotzdem Spaß- auch im Team!

5.2. Psychische Belastung (*Psych*)

Aussage
Überlege mir seit kurzem, - da auch meine Mitarbeiterin im Burn Out ist , - wie es weitergehen soll. Durch zusätzliche belastungen (TI/ telematik Implementierungen , immer mehr Bürokratie und zu viele wechselnde Vorgaben) denke bereits über AUFGABE der Praxis nach !

6. Sonstiges

Aussage
Es fehlt eine Differenzierung zwischen der Rolle der 'Regierung' und der ärztlichen Körperschaften
Interessant wäre eine (teilweise) Wiederholung der Befragung, wenn noch häufiger Corona-Impfungen in Impfzentren bzw. in der hausärztlichen Praxis zusätzlich zum normalen Praxisbetrieb laufen müssen. Die organisatorischen und zeitlichen PRobleme dürften dann massiv zunehmen !!
Antwort ' weiß nicht' bei den Fragen zu Anstrichzentren, da wir selber getrennt von normalen Praxisbetrieb Testungen in großer Zahl vornehmen
es könnten etwas weniger Fragen sein! die Ergebnisse dieser Auswertung würde ich sehr gerne auch erhalten
Ich bitte um Zusendung der Ergebnisse der Umfrage.
Würde gerne über die Ergebnisse der Studie informiert.
Es würde mich interessesieren wie diese Umfrage ausgefallen ist.
Die Frage ob Patienten später behandelt wurden wegen der Corona Triage ist für mich unklar . Sind ein bis 3 Stunden bis max. 1 TAg später relevant später. Ich habe es so aufgefaßt und beantwortet, obwohl ich es als nicht relevant empfinde und sic hbei keinem Patienten ein Nachteil ergeben hat
Ich führe eine private Landarztpraxis und bin nicht vergleichbar mit einem MVZ. Das ist ein großer Unterschied. Hierzu konnte ich keine differenzierten Fragen finden. LG
Fragen teils zu eng gefasst. Es zeigt sich universitäre Distanz zum Praxisbetrieb... trotzdessen die Umfrage von einer unserer Abteilungen gemacht worden ist..!?
Manche Fragestellung war unverständlich
Wie soll eine Zahl für den Patientenstamm abgegeben werden? Alle in der Datenbank? wohl kaum - spiegelt ja nicht das Patientenaufkommen wider. Scheinzahl wäre besser.
manche Fragen hätten man mit 'kommt nicht vor' beantworten könne müssen. so habe ich halt geschrieben was ich getan hätte falls es passiert wäre

..ein wenig lang der Fragebogen,teilweise ungenau formuliert, der Sinn der Frage hat sich manchmal nicht erschlossen
Die Frage mit der Überprüfung habe ich wörtlich genommen
die umfrage hat gar keinen zusammenhang mit corona, sondern zielt auf eine arbeitplatzumfrage /analyse hinsichtlich allgemeinmedizin/migration
die Frage nach der Patientenanzahl ist komplett für mich unklar, wir haben ca 2.300 bis 2.400 Scheine pro Quartal. Daher habe ich mit 0000 ausgefüllt.
Bitte im Anschreiben Abkürzungen vermeiden z B PSA ! Sie können nicht immer davon ausgehen, dass alle Ihre Abkürzungen auch verstehen
Befragung war etwas zu lang, habe mehrfach überlegt, abzubrechen
Gut angelegte Studie
Gut, dass Sie diese Aspekte von COVID beleuchten
Vielen Dank - interessante Fragen für mich zur Reflektion.
Das Infektionsgeschehen entspricht meiner Meinung nach nicht der kommunizierten Schwere
Ich bin froh, in meinem Land arbeiten zu dürfen - wenn ich die Situation in anderen Ländern sehe ...
Ich nehme an, dass mit gesetzliche Vorgangen eigentlich KV Vorgabengemient sind?BZW Abrechungsziffen und -wege. Ich bin schließlich selbstständig und kann alles so machen wie ich es will.
separater Arztkoffer fand ich eine Anregung, wobei wir alle kontaminierten Geräte (Stethoskop usw.) desinfizieren.
Wir haben keiennonline Sprechstunde, dafür eine reine Termin Sprechstunde auch Schon vor corona .... habe versucht das zu übertragen! Beste Unterstützung kam von degam benefits
Wir sind seit 1 Jahr Covid-Schwerpunktpraxis. Ich bin dort die leitende Infektärztin.

7. Index

Änderungen 1, 2, 3, 5, 9

Beruf 5

Einfluss 1, 2, 3

Entscheidungen 3

Experten 6

Gesundheitsversorgung 4

Hausärzte 2, 3, 5, 7

Hausarztpraxis 2, 7

Herausforderung 5

Hygienestationen 7

Impfung 1, 2, 3, 5, 7

Kassenärztlichen Vereinigungen 2, 3, 5

Kollegen 6

Kommunikation 1, 3, 4, 6

Krankenkassen 3

KV 1, 2, 3, 5, 9, 11

Lockdown 2, 5

Mangel 6

Organisation 1, 7

Politik 1, 2, 3, 4, 5, 6, 7

Praxis 1, 2, 3, 6, 7, 9, 10

Privatleben 1, 2, 3, 9

Qualität 1, 4

Rolle 1, 2, 3, 5, 6, 10

Routine 2, 10

Team 2, 3, 5, 9

Unterstützung 1, 2, 4, 11

Versorgung 1, 4, 5

Vorgaben 2, 3, 4, 9

Würdigung 5
