# Supplementary material for: “What else to say?”–Primary health care in times of COVID-19 from the perspective of German general practitioners: An exploratory analysis of the open text field in the PRICOV-19 study
Source: PLoS One. 2023 Mar 17;18(3):e0282504. doi: 10.1371/journal.pone.0282504 (PMC10022805; doi:10.1371/journal.pone.0282504)
Supplement: S3 File — (PDF) [file pone.0282504.s003.pdf]

Häufigkeiten

Hinweise

Ausgabe erstellt		08-JUN-2022 08:59:45
Kommentare		
Eingabe	Daten	V:\Forschung\02 Eigenprojekte\31 Pricov_19\08 Publikationen\02 Pricov Quali STA MK\03 Einreichung\S4 File.sav
	Aktiver Datensatz	DataSet1
	Filter	<keine>
	Gewichtung	<keine>
	Aufgeteilte Datei	<keine>
	Anzahl der Zeilen in der Arbeitsdatei	349
Behandlung fehlender Werte	Definition von fehlenden Werten	Benutzerdefinierte fehlende Werte werden als fehlend behandelt.
	Verwendete Fälle	Statistik basiert auf allen Fällen mit gültigen Daten.
Syntax		FREQUENCIES VARIABLES=feedback /STATISTICS=STDDEV MINIMUM MAXIMUM MEAN MEDIAN SUM SKEWNESS SESKEW /ORDER=ANALYSIS.
Ressourcen	Prozessorzeit	00:00:00,09
	Verstrichene Zeit	00:00:00,09

```
[DataSet1] V:\Forschung\02 Eigenprojekte\31 Pricov_19\08 Publikationen\02 Pricov Quali
 STA MK\03 Einreichung\S4 File.sav
```

Statistiken

Lastly, we would like to hear if you have any additional comments or suggestions for us. All feedback is welcome and can be ente

N	Gültig	349
	Fehlend	0

Lastly, we would like to hear if you have any additional comments or suggestions for us. All feedback is welcome and can be entered in the text box if you wish.

		Häufigkeit	Prozent	Gültige Prozente	Kumulierte Prozente
Gültig		264	75,6	75,6	75,6
	..ein wenig lang der Fragebogen,teilweise ungenau formuliert, der Sinn der Frage hat sich manchmal nicht erschlossen	1	,3	,3	75,9
	Als HÄ erleben wir eine Diskrepanz zwischen Aussagen,Zahlen,Handeln der Politik und Erleben an der Front.die psych/physisch., sozial. Schäden va.der Jungen sind erschreckend,die Massnahmen unverhältnismäßig. Stat. zahlen und Realität Klaffen auseinander.mehr ges.menschenverstand und mut zum vorangehen, Optimismus ,weniger Angstmache und alle Kräfte für Impfungen mobilisieren wäre mein wunsch	1	,3	,3	76,2

Lastly, we would like to hear if you have any additional comments or suggestions for us. All feedback is welcome and can be entered in the text box if you wish.

		Häufigkeit	Prozent	Gültige Prozente	Kumulierte Prozente
	Am Anfang der Pandemie habe ich die Arbeit durch die völlig ungewohnte Situation trotz weniger Patienten als sehr belastend empfunden. Das hat sich im Laufe des Jahres mit Entwicklung einer gewissen Routine gebessert. Das Privatleben leidet deutlich mehr als das Berufsleben unter dem Lockdown. Ich wünsche mir sehr eine baldige Impfung und finde es äußerst seltsam, dass Hausärzte von der Politik nicht in die erste Prioritätsstufe aufgenommen wurden. Wir werden nur deshalb früher geimpft, damit wir die Altenheimpatienten nicht anstecken, aber nicht, damit wir nicht krank werden. Man kann wohl auch ganz gut ohne uns Hausärzte auskommen?!? Dieser Aspekt macht mich am Ende meiner Berufslaufbahn dann schon traurig.	1	,3	,3	76,5
	Am Anfang der Pandemie war die Unterstützung durch die Regierung z.B. durch Schutzkleidung nicht ausreichend. Dies hat sich deutlich gebessert.	1	,3	,3	76,8
	Am stärksten belastet, das jetzt wo eine Impfung verfügbar ist, die Hausärzte, die Heime versorgen, nicht flächendeckend priorisiert werden	1	,3	,3	77,1

Lastly, we would like to hear if you have any additional comments or suggestions for us. All feedback is welcome and can be entered in the text box if you wish.

		Häufigkeit	Prozent	Gültige Prozente	Kumulierte Prozente
	Antwort ' weiß nicht' bei den Fragen zu Anstrichzentren, da wir selber getrennt von normalen Praxisbetrieb Testungen in großer Zahl vornehmen	1	,3	,3	77,4
	Ärger über die sehr schlechte Organisation der Impfungen generell und speziell für zuhause versorgte alte Pat	1	,3	,3	77,7
	Befragung war etwas zu lang, habe mehrfach überlegt, abzubrechen	1	,3	,3	77,9
	Bitte im Anschreiben Abkürzungen vermeiden z B PSA ! Sie können nicht immer davon ausgehen, dass alle Ihre Abkürzungen auch verstehen	1	,3	,3	78,2
	Bitte keine 3. Welle - Impfung für Alle ohne Priorisierung so schnell wie möglich durch die Hausärzte	1	,3	,3	78,5
	Covid war die Chance für viele organisatorische und strukturelle Verbesserungen in der ambulanten Versorgung, die wir künftig weiter verfolgen möchten. Ein lethargiegefährtes Versorgungssystem wurde aufgerüttelt, hat sich mehr auf seine Kernaufgaben besonnen und sich um digitalen Fortschritt bemüht. Die Qualität der Gesundheitsversorgung hat durch die Pandemie gewonnen.	1	,3	,3	78,8

Lastly, we would like to hear if you have any additional comments or suggestions for us. All feedback is welcome and can be entered in the text box if you wish.

		Häufigkeit	Prozent	Gültige Prozente	Kumulierte Prozente
	Das durch die Politik hervorgerufene Chaos und die andauernden Änderungen der Vorgaben haben den täglichen Umgang mit den Patienten und dem Praxisgeschehen mehr beeinflusst als die COVID 19 Pandemie. Die auch heute noch bestehende Hilflosigkeit der Politik nimmt mehr negativen Einfluss auf das Gesundheitssystem als die COVID 19 Pandemie. Mit freundlichen Grüßen vom Lande	1	,3	,3	79,1
	Das Infektionsgeschehen entspricht meiner Meinung nach nicht der kommunizierten Schwere	1	,3	,3	79,4
	das schlimmste ist die KV mit tausenden Regelungen bzgl. der Abrechnung und Überweisung der Abstriche, extrem kompliziert	1	,3	,3	79,7

Lastly, we would like to hear if you have any additional comments or suggestions for us. All feedback is welcome and can be entered in the text box if you wish.

		Häufigkeit	Prozent	Gültige Prozente	Kumulierte Prozente
	Der Umgang mit der Pandemie gehört zu meinem Beruf, das bedeutet Mehrarbeit und starke Fokussierung auf ein Thema, durch den Wegfall vieler Termine im Lockdown allerdings auch zu mehr Familienzeit, insbesondere im 1. Lockdown, die ich sehr geschätzt habe. Ich bin dankbar dafür, recht unbeeinträchtigt weiterarbeiten zu können im vergleich zu anderen Berufsgruppen. Ich leiste meinen Anteil an der Bewältigung der Pandemie und gebe unseren Patienten wenn möglich Halt und Ruhe.	1	,3	,3	79,9
	Die Bundesregierung und Länderregierungen sollten nicht den Rat einzelner selbstgewählter Experten befolgen, sondern ein vorgegebenes Gremium aus verschiedenen Experten hören. Das Grundgesetz und die parlamentarische Demokratie darf nicht so einfach Außerkraft gesetzt werden können. Der Gesundheitsminister sollte zwangsweise Mediziner aus verschiedenen Sparten in seinem Ministerium enthalten haben und konsultieren müssen.	1	,3	,3	80,2
	Die Frage mit der Überprüfung habe ich wörtlich genommen	1	,3	,3	80,5

Lastly, we would like to hear if you have any additional comments or suggestions for us. All feedback is welcome and can be entered in the text box if you wish.

		Häufigkeit	Prozent	Gültige Prozente	Kumulierte Prozente
	die Frage nach der Patientenanzahl ist komplett für mich unklar, wir haben ca 2.300 bis 2.400 Scheine pro Quartal. Daher habe ich mit 0000 ausgefüllt.	1	,3	,3	80,8
	Die Frage nach der Patientenpopulation (Zahl der Patienten), aber 'NICHT-Scheinzahl' ist für mich als Vertragsarzt schwer zu verstehen; die Patientennummer unserer elektronischen Kartei steht bei 14500. Wir versorgen eine Apalliker-Beatmungseinrichtung (max. 34 Betten), ein gerontopsychiatrisches Heim (nicht immer geronto..., leider auch immer wieder Jüngere; und immer palliative Patienten) und eine Behinderten-Wohneinrichtung mit drei Einheiten - insofern dürften wir eine nicht besonders typische Praxis sein; und fühlten uns in der Pandemie deswegen auch und gerade besonders im Stich gelassen und als Ärzte bzw. Praxisteam zweiter Klasse!	1	,3	,3	81,1
	Die Frage ob Patienten später behandelt wurden wegen der Corona Triage ist für mich unklar . Sind ein bis 3 Stunden bis max. 1 TAg später relevant später. Ich habe es so aufgefaßt und beantwortet, obwohl ich es als nicht relevant empfinde und sic hbei keinem PAtienten ein Nachteil ergeben hat.	1	,3	,3	81,4

Lastly, we would like to hear if you have any additional comments or suggestions for us. All feedback is welcome and can be entered in the text box if you wish.

	Häufigkeit	Prozent	Gültige Prozente	Kumulierte Prozente
Die Information der Politik über die Medien und das was ich in meiner Praxis umsetzen kann stimmte zu keiner Zeit über ein. Es werden Entscheidungen getroffen, die ich nicht umsetzen kann, weil Material oder die Voraussetzungen fehlen. So jetzt wieder geschehen mit den Schnelltestung in meiner Praxis. Wie soll ich hier jedem einen Schnell Test, der es wünscht anbieten können wo ich zum einen drauf bezahle, so viel schnelltest nicht habe, Infektionssprechstunde habe und kranke behandle. Es ist nicht auf meine Aufgabe gesunde Menschen mit schnelltest zu versorgen -z bsp. Die fast wöchentlich Änderung der Abrechnungsmodalitäten bezüglich COVID im Herbst 2020 (Lehrer, Reise Rückkehrer, Kontakt Personen etc.) war gelinde gesagt eine Zumutung und fast nicht umsetzbar. Die Flut an wöchentlichen, ja täglichen Informationen und der Unsinn der in der Presse bezüglich COVID verbreitet wird belastet mich mehr als die Behandlung und der Umgang mit den Patienten.	1	,3	,3	81,7

Lastly, we would like to hear if you have any additional comments or suggestions for us. All feedback is welcome and can be entered in the text box if you wish.

		Häufigkeit	Prozent	Gültige Prozente	Kumulierte Prozente
	Die laufenden KV Mitteilungen und vielen Änderungen im Corona Procedere und der Kampf um Desinfektionsmittel etc nervten. Dabei war nicht mehr soviel Zeit wie vorher für andere Weiterbildungen. Die Patienten sind zunehmend gereizter. Die Schwestern haben noch den Zweitjob Homeschooling, der zusätzlich Energie frisst. Das Leben macht trotzdem Spaß- auch im Team!	1	,3	,3	81,9
	Die politische Koordination ist eine absolute Katastrophe, keine Linie, keine entsprechende Kompetenz, alles nur wirr und jeder macht was anderes. Es geht um die nächste Wahl. Es existiert für niemanden ein verlässlicher Leitfaden	1	,3	,3	82,2
	Die rolle der Hausärzte ist nicht stark genug. Wir haben 90% der Covid-Patienten versorgt und sollten noch nicht einmal in der ersten Impfgruppe eine Impfung bekommen. das ist sehr traurig und frustrierend. Sich hejden Tag in Lebensgefagr begeben und dafür mit Spätimpfung abgestraft zu werden, ist eine Katastrophe. Tägliche Änderungen seitens der Kassenärztlichen Vereinigungen waren in dem Tempe insbesondere mit Teaching der Mitarbietriennen kaum umzusetzen.	1	,3	,3	82,5

Lastly, we would like to hear if you have any additional comments or suggestions for us. All feedback is welcome and can be entered in the text box if you wish.

		Häufigkeit	Prozent	Gültige Prozente	Kumulierte Prozente
	die umfrage hat gar keinen zusammenhang mit corona, sondern zielt auf eine arbeitplatzumfrage /analyse hinsichtlich allgemeinmedizin/migration	1	,3	,3	82,8
	Eine große Entlastung für alle Mitarbeitenden in unserer Praxis wäre eine baldige Impfung!	1	,3	,3	83,1
	Erbarmungswürdig ist die Unwissenheit der Menschen auch nach einem Jahr Pandemie. Der Staat als Aufklärer hat versagt (Bewegung, Vitamine, Maskenkunde). Die Kommunikation erhält eine glatte fünf.	1	,3	,3	83,4

Lastly, we would like to hear if you have any additional comments or suggestions for us. All feedback is welcome and can be entered in the text box if you wish.

		Häufigkeit	Prozent	Gültige Prozente	Kumulierte Prozente
	Es fehlt die Frage ob die Kollegen sich selber auf die Pandemie vorbereitet haben. wir sind nicht in der Schule dass die Regierung alles machen für uns muss. Man sollte auch selbst die Zeichen der Zeit lesen können. Als ich mit Schutzkleidung Faceshield und Maske am 23.2..2020 in whatsapp auftrat erntete ich einen Shitsturm . Am 1.3. hatten wir Masken, Trennwände und Schutzkleidung wie Triage und extra Sprechzeiten für Patienten mit Infekten. Viele Kollegen haben zu dieser Zeit noch geträumt. Keiner meiner Angestellten mit Familie waren je krank, wir arbeiten mehr als vorher aber wir sind stolz als Testpraxis und Versorgungspraxis unsere Gemeinde und Umgebung unterstützt zu haben Wir haben koronabedingt 1/3 mehr Patienten, aber das wird sich wieder geben.Wir hatten nie einen Mangel an Masken, Desinfektionsmaterial oder Schutzkleidung für die Praxis, unser Personal oder unsere Familien	1	,3	,3	83,7
	Es fehlt eine Differenzierung zwischen der Rolle der 'Regierung' und der ärztlichen Körperschaften	1	,3	,3	84,0
	es gibt zu wenige Informationen über die ambulante COVID-Therapie	1	,3	,3	84,2

Lastly, we would like to hear if you have any additional comments or suggestions for us. All feedback is welcome and can be entered in the text box if you wish.

		Häufigkeit	Prozent	Gültige Prozente	Kumulierte Prozente
	es könnten etwas weniger Fragen sein! die Ergebnisse dieser Auswertung würde ich sehr gerne auch erhalten	1	,3	,3	84,5
	Es wäre schön, wenn die zuständigen Stellen nicht so überbürokratisiert wären und zB Abrechnung etc EINFACH statt zT 8-fach erledigt werden könnte.	1	,3	,3	84,8
	Es wird zeit, Dass hHausärzte überhaupt wahrgenommen werden von Politik und Administration. Wir könnten schon längst ins Impfen eingebunden sein. Aber es gibt noch nicht einmal Informationen, wie dies geschehn wird, wenn die Impfstoffwelle anrollt. Die Rolle der Ärztekammer ist erbärmlich. Die Kommunikation mit uns Ärzten auch.	1	,3	,3	85,1
	Es würde mich interessesieren wie diese Umfrage ausgefallen ist. LG T. Kehler	1	,3	,3	85,4
	Fragen teils zu eng gefasst. Es zeigt sich universitäre Distanz zum Praxisbetrieb... trotzdessen die Umfrage von einer unserer Abteilungen gemacht worden ist..!?	1	,3	,3	85,7
	Gut angelegte Studie	1	,3	,3	86,0

Lastly, we would like to hear if you have any additional comments or suggestions for us. All feedback is welcome and can be entered in the text box if you wish.

		Häufigkeit	Prozent	Gültige Prozente	Kumulierte Prozente
	Gut, dass sie das machen. Hoffentlich wacht die Politik dann auf. Viel Hoffnung habe ich diesbezüglich allerdings nicht. Die Hausarztmedizin wird seit eh und jeh stark vernachlässigt, nicht gewürdigt und völlig unterbezahlt (jedenfalls im KV-Bezirk Hamburg). Daran wird auch die Pandemie und deren Bekämpfung nichts ändern, im Gegenteil.	1	,3	,3	86,2
	Gut, dass Sie diese Aspekte von COVID beleuchten	1	,3	,3	86,5

Lastly, we would like to hear if you have any additional comments or suggestions for us. All feedback is welcome and can be entered in the text box if you wish.

		Häufigkeit	Prozent	Gültige Prozente	Kumulierte Prozente
	Habe die Praxis im April 2020 übernommen und fühle mich durch KV nicht unterstützt. Profitierte nicht vom Rettungsschirm der KV obwohl Patienteneinbruch wie alle Praxen. Sie berechnen meinen Rettungsschirm nicht mit 917 -1000 wie Vorgängerin hatte sondern 719 Patienten, welche wir nie unterschreiten werden. Einen Praxisabgang von Patienten gab es kaum da unterversorgtes Gebiet. Ich habe aber durch die Modernisierung und gestiegenen Preise fürHygiene mehr Ausgaben als meine Abgeberin. Die erste Abschlagszahlung lag bei 7000 € anstelle 14000 weil es die KV Berlin Vorstände so beschlossen hatten für Neu übernommene Praxen. Kita Notbetreuung funktionierte auch nicht und musste privat bezahlt werden.	1	,3	,3	86,8
	Ich bin froh, in meinem Land arbeiten zu dürfen - wenn ich die Situation in anderen Ländern sehe ...	1	,3	,3	87,1
	Ich bitte um Zusendung der Ergebnisse der Umfrage.	1	,3	,3	87,4

Lastly, we would like to hear if you have any additional comments or suggestions for us. All feedback is welcome and can be entered in the text box if you wish.

		Häufigkeit	Prozent	Gültige Prozente	Kumulierte Prozente
	Ich fand die unglaubliche Informationsflut mit den vielen, sehr schnellen Änderungen (denen wir als Kassenärzte im letzten Jahr ausgesetzt waren und auf die wir immer blitzschnell reagieren mussten) im letzten Jahr sehr belastend!	1	,3	,3	87,7
	ich finde die Coroanzeit demaskierend für die soziale Einstellung der einzelnen Team-Mitglieder. Über die Bereitschaft der allermeisten die Ärmel hoch zu krämpeln und anzupacken bin ich sehr dankbar. Letzendlich sind wir als Team zusammen gerückt	1	,3	,3	88,0
	Ich führe eine private Landarztpraxis und bin nicht vergleichbar mit einem MVZ. Das ist ein großer Unterschied. Hierzu konnte ich keine differenzierten Fragen finden. LG	1	,3	,3	88,3
	Ich mache mir finanzielle Sorgen. Steigende Personalkosten (MFA-Tarifvertrag) und sinkende Einnahmen (Patientenzahlen) verursachen aktuell eine neue finanzielle 'Schieflage'.	1	,3	,3	88,5
	Ich nehme an, dass mit gesetzliche Vorgangen eigentlich KV Vorgabengemient sind? BZW Abrechungsziffen und -wege. Ich bin schließlich selbstständig und kann alles so machen wie ich es will.	1	,3	,3	88,8

Lastly, we would like to hear if you have any additional comments or suggestions for us. All feedback is welcome and can be entered in the text box if you wish.

		Häufigkeit	Prozent	Gültige Prozente	Kumulierte Prozente
	Ich sehe die Pandemie als große Gefahr und Herausforderung. Aber ich sehe es auch als grundsätzliche ärztlich Aufgabe sich dieser Herausforderung zu stellen.	1	,3	,3	89,1
	ich versorge ,unter anderem, 80 Opiatsubstituierte, diese Gruppe hatte unter dem lockdown sehr zu leiden, da Hilfesysteme schlossen und Obdachlose keine Unterkünfte mehr hatten und kein Essen mehr ausgegeben wurde, das fand ich menschenunwürdig	1	,3	,3	89,4
	Ihr Fragebogen hat nicht die Praxen eingeschlossen/angesprochen, die selbst durch Umbaumaßnahmen eine Trennung von Infizierten und 'normalen' potentiell nicht Infizierten Patienten vorgenommen hat. Wir haben einen Container aufgebaut draußen, vor der Praxis und machen selber Abstriche sowie Diagnostik und Therapie aller Infizierten - im Container. Das schützt die 'normalen' Patienten und gibt denen auch Zuversicht, zum Arzt gehen zu können.	1	,3	,3	89,7
	In Phasen des Lockdowns ist das Patientenaufkommen in der Hausarztpraxis deutlich rückläufig mit erheblichen finanziellen und wirtschaftlichen Risiken für die Praxen.	1	,3	,3	90,0

Lastly, we would like to hear if you have any additional comments or suggestions for us. All feedback is welcome and can be entered in the text box if you wish.

		Häufigkeit	Prozent	Gültige Prozente	Kumulierte Prozente
	Interessant wäre eine (teilweise) Wiederholung der Befragung, wenn noch häufiger Corona-Impfungen in Impfzentren bzw. in der hausärztlichen Praxis zusätzlich zum normalen Praxisbetrieb laufen müssen. Die organisatorischen und zeitlichen PRobleme dürften dann massiv zunehmen !!	1	,3	,3	90,3
	keine Kommentare	1	,3	,3	90,5
	Leider drängen sich immer mehr fachfremde Instanzen in unser Berufsbild und bevormunden. Krankenkassen versuchen zunehmend insbesondere Hausärzte zu denunzieren und Patienten vom Arzt abzudrängen.	1	,3	,3	90,8
	manche Fragen hätten man mit 'kommt nicht vor' beantworten könne müssen. so habe ich halt geschrieben was ich getan hätte falls es passiert wäre	1	,3	,3	91,1
	Manche Fragestellung war unverständlich	1	,3	,3	91,4

Lastly, we would like to hear if you have any additional comments or suggestions for us. All feedback is welcome and can be entered in the text box if you wish.

		Häufigkeit	Prozent	Gültige Prozente	Kumulierte Prozente
	Mein großer Wunsch an die Politik: ärztliche Telefonate auch nach der Pandemie zu vergüten, denn 4h/d gratis zu telefonieren, ist unseres Berufstandes unwürdig. Durch die Telefonate können Ansteckungsmöglichkeiten in den Praxen dtl. reduziert werden und die Patienten sind trotzdem gut versorgt. Corona per se hat meine Arbeitsbelastung nicht erhöht (der bürokratische Mehraufwand und die verminderte Patientenzahl halten sich die Waage).	1	,3	,3	91,7
	mit Ruhe und Zuversicht werden wir die Pandemie überstehen! DIe Flut an Informationen durch verschiedene Stellen ist sehr anstrengend! Kann man leicht den Überblick und die Aufmerksamkeit verlieren !	1	,3	,3	92,0
	nein	2	,6	,6	92,6
	Nein	2	,6	,6	93,1
	Praxis ließ sich nur so gut organisieren, weil wir über einen separaten Seminarraum verfügen, der gegenüber in einem anderen Gebäude liegt, und zur Infektsprechstunde genutzt werden kann.	1	,3	,3	93,4

Lastly, we would like to hear if you have any additional comments or suggestions for us. All feedback is welcome and can be entered in the text box if you wish.

		Häufigkeit	Prozent	Gültige Prozente	Kumulierte Prozente
	Praxisübernahme seit 2021, daher teilweise Antworten unter Bezugnahme auf bestehende Prozesse erforderlich. Durch die offiziellen Stellen (KV, ÄK, HZV) wenig Vorbereitung/UNterstützung für die erschwerte Situation einer Unternehmensgründung unter Pandemieeinflüssen, insbesondere im ländlichen Raum mit erheblichen Strukturdefiziten.	1	,3	,3	93,7
	Problematisch war neben Covid das Thema Impfungen. Die Grippeimpfung war seitens unserer Patienten stark erwünscht, Impfstoff kam zu wenig zu spät. Pneumokokken 23 Impfung ist für uns seit über 1 Jahr nicht verfügbar. Ich befürchte, dass die Covid-Impfungen nach entsprechender Zulassung tauglicher Impfstoffe zu früh von der Politik auf die Hausarztpraxen rückdelegiert werden und wir mangels ausreichender Impfstoffmengen wieder unseren Patienten gegenüber, ohne dass wir etwas dafür können, in Erklärungsnot geraten... let's see what happens...	1	,3	,3	94,0

Lastly, we would like to hear if you have any additional comments or suggestions for us. All feedback is welcome and can be entered in the text box if you wish.

		Häufigkeit	Prozent	Gültige Prozente	Kumulierte Prozente
	Sehr belastend sind die täglich - wöchentlich sich ändernden Vorgaben für Abrechnung, Testungen , sowie die Kommunikation mit dem Gesundheitsamt. Dies hat um mind.1/3 zugenommen und bindet sehr viel Kraft und Zeit, die dann für den direkten Patientenkontakt fehlt .	1	,3	,3	94,3
	Seit COVID hat sich unsere Praxis mit der Nachbarpraxis die Infektsprechstunde geteilt, sodass wir nur an 3 Nachmittagen in der Woche Infektsprechstunde hatten und auch so Zeit für die Impfungen und Check ups finden konnten.	1	,3	,3	94,6
	separater Arztkoffer fand ich eine Anregung, wobei wir alle kontaminierten Geräte (Stethoskop usw.) desinfizieren.	1	,3	,3	94,8
	Sind Corona-Abstrich-Praxis.Haben Zelte vor der Praxis aufgebaut um dort Triade und Abstriche zu machen.Trennung zwischen Infektsprechstunde und anderen Erkrankungen	1	,3	,3	95,1
	Strikte Trennung der Patienten: Infektionssprechstunde zu Extra- Zeiten in nur dafür genutztem Raum außerhalb der normalen Praxis	1	,3	,3	95,4

Lastly, we would like to hear if you have any additional comments or suggestions for us. All feedback is welcome and can be entered in the text box if you wish.

		Häufigkeit	Prozent	Gültige Prozente	Kumulierte Prozente
	Überlege mir seit kurzem, - da auch meine Mitarbeiterin im Burn Out ist , - wie es weitergehen soll. Durch zusätzliche belastungen (TI/ telematik Implementierungen , immer mehr Bürokratie und zu viele wechselnde Vorgaben) denke bereits über AUFGABE der Praxis nach !	1	,3	,3	95,7
	Unterstützung während der Pandemie kam vom Hausärzteverband mit praktischen Tipps	1	,3	,3	96,0
	Unzureichende Würdigung in finanzieller und politischer Hinsicht Rolle und Aufgaben der Hausärzte, fehlende Mitbeteiligung Impfung Covid-19	1	,3	,3	96,3
	Vielen Dank - interessante Fragen für mich zur Reflektion.	1	,3	,3	96,6
	Warum waren Hausärzte und Team nicht von Beginn an in Gruppe 1 für die Corona-Impfungen. Wir waren im März 2020 ein Hotspot und haben uns bis jetzt nicht erholt. Die Praxis droht zu zerfallen! 3 Vollzeitkräfte sind gegangen! Bisher waren wir immer ein tolles Team und bestehen seit 34 Jahren als große Hausarztpraxis .... Zur Zeit weiß ich nicht, wie es weitergehen wird.	1	,3	,3	96,8

Lastly, we would like to hear if you have any additional comments or suggestions for us. All feedback is welcome and can be entered in the text box if you wish.

	Häufigkeit	Prozent	Gültige Prozente	Kumulierte Prozente
Was im Fragebogen nicht so richtig rauskommt, ist der disruptive Aspekt der Pandemie bzgl. jeglicher Routine. Es wäre ein spannendes Feld zu hinterfragen, wie Krisen (Pandemie, Klima etc.) sich jetzt und zukünftig auf unsere individualisierte Patientenversorgung auswirkt und mit welchen Werkzeugen, wir das besser integrieren können.	1	,3	,3	97,1
Weniger Pat. oder Krankheiten stressen uns, vielmehr der ständige Wechesel von Bestimmungen, geänderte Anordnungen, Vorgaben etc. Oft erfährt man am Fr. Nachmittag, was sich am Mo. ändern wird, und am Di dann, dass es doch anders gemacht werden sollte... (Bsp. PCR/POC-Tests!)	1	,3	,3	97,4
Wichtig wäre noch die Frage, ob man sich in dieser Zeit genug informiert und unterstützt gefühlt hat, vom öffentlichen Gesundheitswesen, von der KV, von der örtlichen Verwaltung. Das ist bei mir eben gar nicht der Fall: ich weiß auf die meisten Fragen meiner PatientInnen einfach keine vernünftige Antwort.	1	,3	,3	97,7

Lastly, we would like to hear if you have any additional comments or suggestions for us. All feedback is welcome and can be entered in the text box if you wish.

		Häufigkeit	Prozent	Gültige Prozente	Kumulierte Prozente
	Wie soll eine Zahl für den Patientenstamm abgegeben werden? Alle in der Datenbank? wohl kaum - spiegelt ja nicht das Patientenaufkommen wider. Scheinzahl wäre besser.	1	,3	,3	98,0
	Wir haben die Anmeldung dauerhaft umgebaut. Wir haben Hygienestationen an drei Eingängen angebracht. Wir haben vor 2 Jahren unsere neu gebaut Praxis bezogen, die wir zu Zeiten der Schweinegrippe für dieses Szenario geplant haben und so bestens zurecht kommen!	1	,3	,3	98,3
	Wir haben keiennonline Sprechstunde, dafür eine reine Termin Sprechstunde auch Schon vor corona .... habe versucht das zu übertragen! Beste Unterstützung kam von degam benefits	1	,3	,3	98,6

Lastly, we would like to hear if you have any additional comments or suggestions for us. All feedback is welcome and can be entered in the text box if you wish.

		Häufigkeit	Prozent	Gültige Prozente	Kumulierte Prozente
	Wir sind nun besser in der Praxis organisiert, der Zusammenhalt ist besser und die Patienten machen gut mit. Wir haben den Vorteil zwei Eingänge zu haben Ich untersuche alle Covid Patienten allein in einem seperatem Raum, nur ich habe Kontakt mit diesen. Die MFA sind völlig geschützt. Covid 19 hat vieles, so schlimm das auch ist, zum Guten verändert. Auch sind wir schon geimpft, da wir drei AH betreuen und Infektsprechstunden abhalten. Wir versuchen auch jetzt schon uns sehr auf die Coronaimpfungen vorzubereiten und haben die Sprechstunden ab April erweitert 2021 Insgesamt ist die Belastung für eine Einzelarztpraxis sehr hoch weil die Quarantäne bis zur Impfung über uns schwebte, das war das schlimmste. Es wird schon	1	,3	,3	98,9
	Wir sind seit 1 Jahr Covid-Schwerpunktpraxis. Ich bin dort die leitende Infektärztin.	1	,3	,3	99,1

Lastly, we would like to hear if you have any additional comments or suggestions for us. All feedback is welcome and can be entered in the text box if you wish.

		Häufigkeit	Prozent	Gültige Prozente	Kumulierte Prozente
	Wir würden uns wünschen, daß zum einen MFA's einen Covid-Zuschlag erhalten, so wie es der Hausarztverband schon lange fordert. Und zum anderen wäre es motivierend, wenn auch die Politik die Leistungen der ambulanten Versorgungsebene und hier insbesondere die hausärztliche Versorgung mehr wertgeschätzt würde.	1	,3	,3	99,4
	Würde gerne über die Ergebnisse der Studie informiert.	1	,3	,3	99,7
	Zuviel zu lesen, Erlasse zu viel und zu spät	1	,3	,3	100,0
	Gesamt	349	100,0	100,0	
